# Fgf8 dynamics and critical slowing down may account for the temperature independence of somitogenesis

**DOI:** 10.1038/s42003-022-03053-0

**Published:** 2022-02-07

**Authors:** Weiting Zhang, Pierluigi Scerbo, Marine Delagrange, Virginie Candat, Vanessa Mayr, Sophie Vriz, Martin Distel, Bertrand Ducos, David Bensimon

**Affiliations:** 1 LPENS, PSL, CNRS, 24 rue Lhomond, 75005 Paris, France; 2 IBENS, PSL, CNRS, 46 rue d’Ulm, 75005 Paris, France; 3 High Throughput qPCR Core Facility, ENS, PSL, 46 rue d’Ulm, 75005 Paris, France; 4 St Anna Children’s Cancer Research Institute, Zimmermannplatz 10, 1090 Vienna, Austria; 5 CIRB, Collège de France, 75005 Paris, France; 6 Department of Chemistry and Biochemistry, UCLA, Los Angeles, 90094 USA

**Keywords:** Embryonic induction, Body patterning

## Abstract

Somitogenesis, the segmentation of the antero-posterior axis in vertebrates, is thought to result from the interactions between a genetic oscillator and a posterior-moving determination wavefront. The segment (somite) size is set by the product of the oscillator period and the velocity of the determination wavefront. Surprisingly, while the segmentation period can vary by a factor three between 20 °C and 32 °C, the somite size is constant. How this temperature independence is achieved is a mystery that we address in this study. Using RT-qPCR we show that the endogenous *fgf8* mRNA concentration decreases during somitogenesis and correlates with the exponent of the shrinking pre-somitic mesoderm (PSM) size. As the temperature decreases, the dynamics of *fgf8* and many other gene transcripts, as well as the segmentation frequency and the PSM shortening and tail growth rates slows down as T–T_c_ (with T_c_ = 14.4 °C). This behavior characteristic of a system near a critical point may account for the temperature independence of somitogenesis in zebrafish.

## Abstract (web summary)

In Zebrafish, the dynamics of fgf8 and other gene transcripts as well as segmentation frequency, shortening of pre-somitic mesoderm and tail growth rate slows down with lower temperature. This may explain the temperature independence of somitogenesis.

## Introduction

Somitogenesis is the process of segmentation of the antero-posterior axis in vertebrates. In zebrafish this process starts at about 10 hpf and ends at 24 hpf^1^. During that developmental interval, as the embryo elongates, pairs of somites synchronously and periodically pinch off from the anterior part of the PSM in an anterior to posterior series until 31 pairs of somites are formed. The period between somites, but surprisingly not their size, is strongly temperature dependent^2^.

**Fig. 1:**
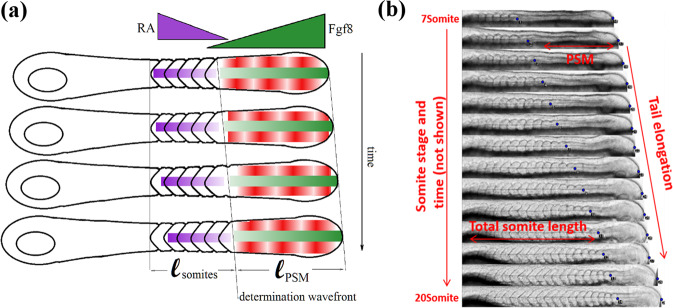
Schematics of somitogenesis. **a** The Clock and Wavefront model: antagonistic gradient of Fgf8 (originating from the posterior PSM, green) and RA (originating from the somites, violet) define a wavefront which interacts with a particular phase of the segmentation clock (in the PSM, red) to generate somites at periodic times and positions. **b** Kymograph of somitogenesis from 7 to 20 somites. The tail elongates at a constant rate *V*_tail_ while the PSM shrinks at a roughly constant rate *V*_PSM_ resulting in a somite wavefront propagating^5^ (in the lab frame) at a rate *V*_front_ = *V*_tail_ − *V*_PSM_.

The formation of somites in zebrafish is preceded by the establishment of a segmental pre-pattern in the anterior PSM accomplished by a stripe of gene expression that is thought to result from the interactions between a genetic oscillator and a posterior-moving determination wavefront. This pre-pattern determines the position of the next somite. The Clock and Wavefront framework first proposed by Cooke and Zeeman^3^ in 1976, is currently used to describe the output of the complex genetic network underlying the formation of this pre-pattern. In this framework, periodic oscillations (the segmentation clock) that move anteriorly (in the PSM reference frame^4^), pass through a determination wavefront moving posteriorly (in both the lab and the PSM reference frames) and stop oscillating. As a result, a stripe of genes such as *mesp2* are activated to establish the future boundary of the following somite^1^. In this model, the size of a somite is determined by the distance traveled by the determination wavefront during one cycle of the segmentation clock^4,5^ (see Fig. 1).

The segmentation clock driving the differentiation of the PSM into somites at the determination wavefront, has been amply studied and described^4,6^ and is not the subject of this work. Rather we here focus on the determination wavefront. In contrast to the segmentation clock whose details are species dependent, the main putative actors of the wavefront (e.g., fibroblast growth factors (Fgf), retinoic acid (RA)) are conserved in vertebrates (from fish to mammals, including snakes and amphibians^7^). Previous studies have shown that as cells exit from the progenitor domain at the posterior end of the PSM, they stop transcribing *Fgf* genes^8–11^. Thus, *fgf* mRNA progressively decays as cells move towards the anterior of the PSM and an *fgf* gradient is formed^12^. This mRNA gradient is translated into a protein gradient and into a MAPK activity gradient along the PSM (as Erk, a MAPK protein is activated downstream off the FGF receptor)^9,10^.

Spurred by these experiments we have decided to investigate the dynamics of the PSM in fish embryos subjected to various perturbations of the putative actors of the wavefront (Fgf, RA, Erk, etc.). For these investigations we used time-lapse and fluorescent microscopy on live wild-type or transgenic embryos expressing either a fluorescent reporter of Erk activity or an exogenous source of Fgf8. In agreement with experiments on tail explants^13^, our results imply that somite formation results from a coupling between the somitogenetic clock and the local spatial (but not temporal) gradient of Fgf8 (or Erk activity). Expanding on these previous investigations, we observe that the PSM size vary as the logarithm of the *fgf8* mRNA concentration, a result that has a simple explanation if the *fgf8* mRNA concentration decays exponentially in both space and time.

In addition, we took advantage of the external development of zebrafish embryos to manipulate the temperature of development. As the temperature T is lowered we find that the dynamics of *fgf8* and many other genes implicated in somitogenesis and cellular differentiation slow down by the same factor, namely as (*T*−*T*_c_) (with *T*_c_ = 14.4 °C). Slowing down by this factor is also observed^2^ for the segmentation frequency f_s_, the spatio-temporal dynamics of Erk and the PSM shrinkage and tail growth rates. These observations suggest that the temperature invariance of the developmental program during somitogenesis could be a simple reflection of the critical slowing down of some cellular metabolic networks near a critical temperature *T*_c_.

## Results

### Kinetics of somitogenesis impairment by Fgf-pathway inhibition

**Fig. 2:**
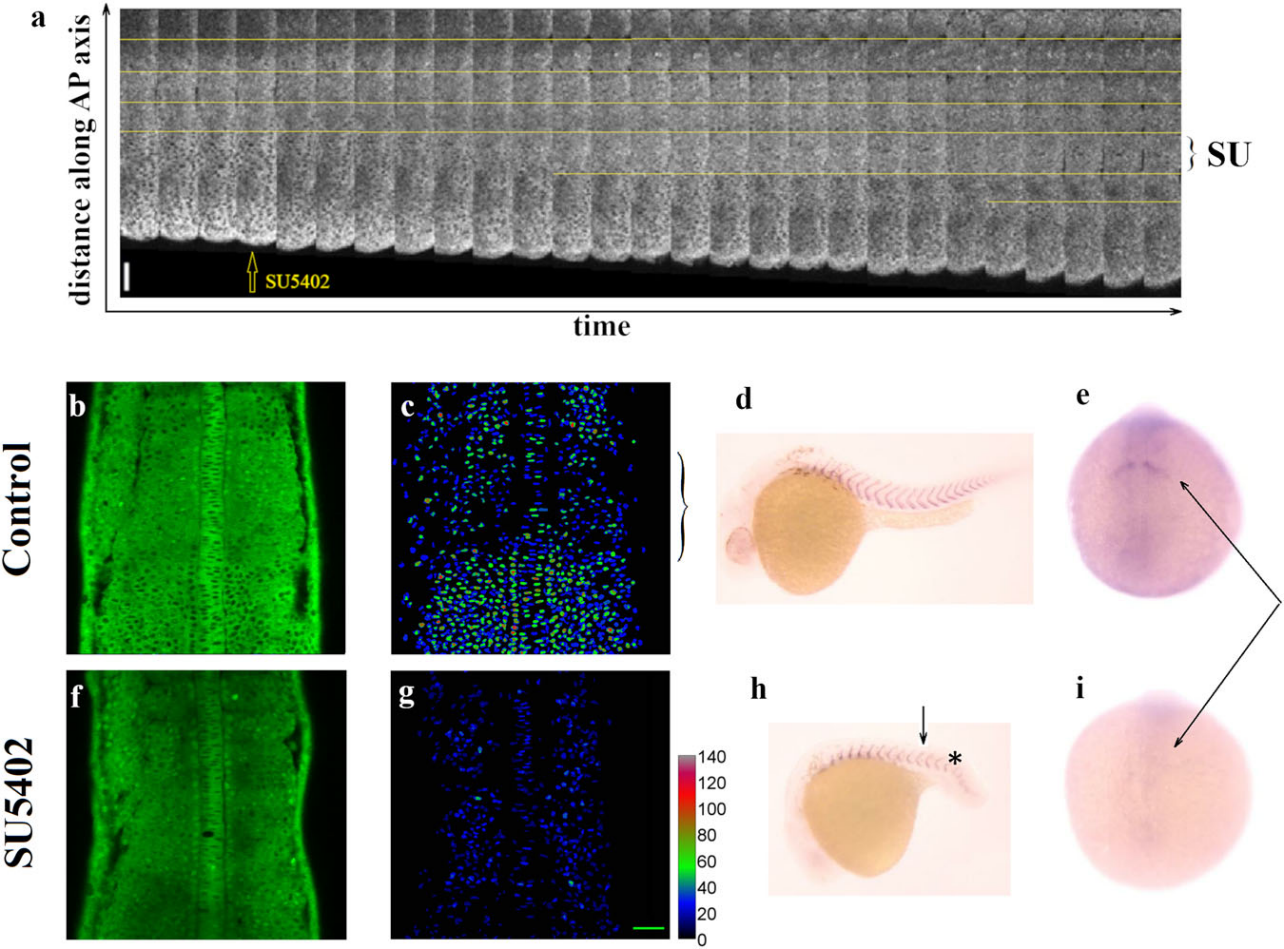
Somitogenesis at low Erk activity. **a** Time lapse observations of the Erk activity domain in DREKA embryos following exposure to a low (50 μM) concentration of SU5402 (an inhibitor of the Fgf pathway). The time span between images is 4 min. (the period of the clock in this experiment is about 45 min.) The yellow lines point to the boundary of Erk activity which periodically jumps by about 50 μm except in the first period after administration of SU5402 where the jump is larger (labeled as SU). Scale bar: 50 μm. **b**–**e** Normal somitogenetic development in DREKA embryos (control). **b** Erk activity in DREKA embryos at 14 somite stage: dark nuclei point to high Erk activity. **c** The false color images represent the Erk activity averaged over a 30 μm perpendicular stack of images. Notice the low activity area in the pre-pattern region (shown by the bracket) at the anterior PSM. **d** In situ hybridization (ISH) against *xirp2a* (a marker of somite boundaries) in 30 hpf embryos. **e** Immuno-Histochemical (IHC) staining against Mesp2a, a determinant of the last somite boundary (arrow). **f**–**i** Somitogenetic development in DREKA embryos in presence of a high (200 μM) concentration of SU5402 from the 10 somite stage (arrow in (h)). Somitogenesis is impaired past 13 somite stage (indicated by a * in **h**) with unclear somite boundaries. **f**, **g** The Erk activity is repressed (low) throughout the PSM. **h** ISH against *xirp2a* in 30 hpf embryos. **i** The determinant of the last somite boundary (Mesp2a), shown by an arrow is absent past 13 somite stage in **i** but present in **e**. Scale bar: 50 μm.

We monitored the dynamics of Erk during somitogenesis in transgenic zebrafish (DREKA) embryos expressing a fluorescent reporter which cytoplasmic localization increases upon increasing Erk phosphorylation^14,15^. Changes in Erk activity (i.e., phosphorylation) are thus well reflected and reported by changes in the ratio between cytoplasmic and nuclear fluorescence (Fig. 2a–c). As previously reported^16–18^ we observed that the domain of Erk activity shrank over time (Fig. S1a, b), with transitions at the segmentation clock period from a high activity to a low activity domain at a distance of about 3–4 somites down from the last somite, i.e., 3–4 segmentation periods prior to the appearance of a new somite (Fig. S1c). This transition in Erk phosphorylation is the earliest indication of commitment to differentiation into somites^16^.

Having confirmed the role of Erk as an early somitic marker, we then studied the response of an embryo to perturbations of the Fgf pathway (of which Erk is a downstream effector) by pharmacological inhibition of the pathway with SU5402. Embryos exposed to a low concentration of SU5402 (50 μM), display an increase in the next jump of the Erk domain of activity (i.e., a larger somite three periods later) (Fig. 2a), followed by regular jumps (and somites) similar to WT embryos^18^. However, if the embryos are exposed (at the ten somites stage) to 200 μM of SU5402 the activity of Erk is completely repressed in all the PSM (compare Fig. 2f, g with the control shown in Fig. 2b, c). In such case, somite formation is impaired three segmentation periods later (i.e., from the 13 somites stage onward) (compare Fig. 2h, i with Fig. 2d, e); regular somites are not observed and expression of Mesp2a (a marker of the last somite^1^) is absent.

### Uniform activation of the Fgf pathway impairs somitogenesis

**Fig. 3:**
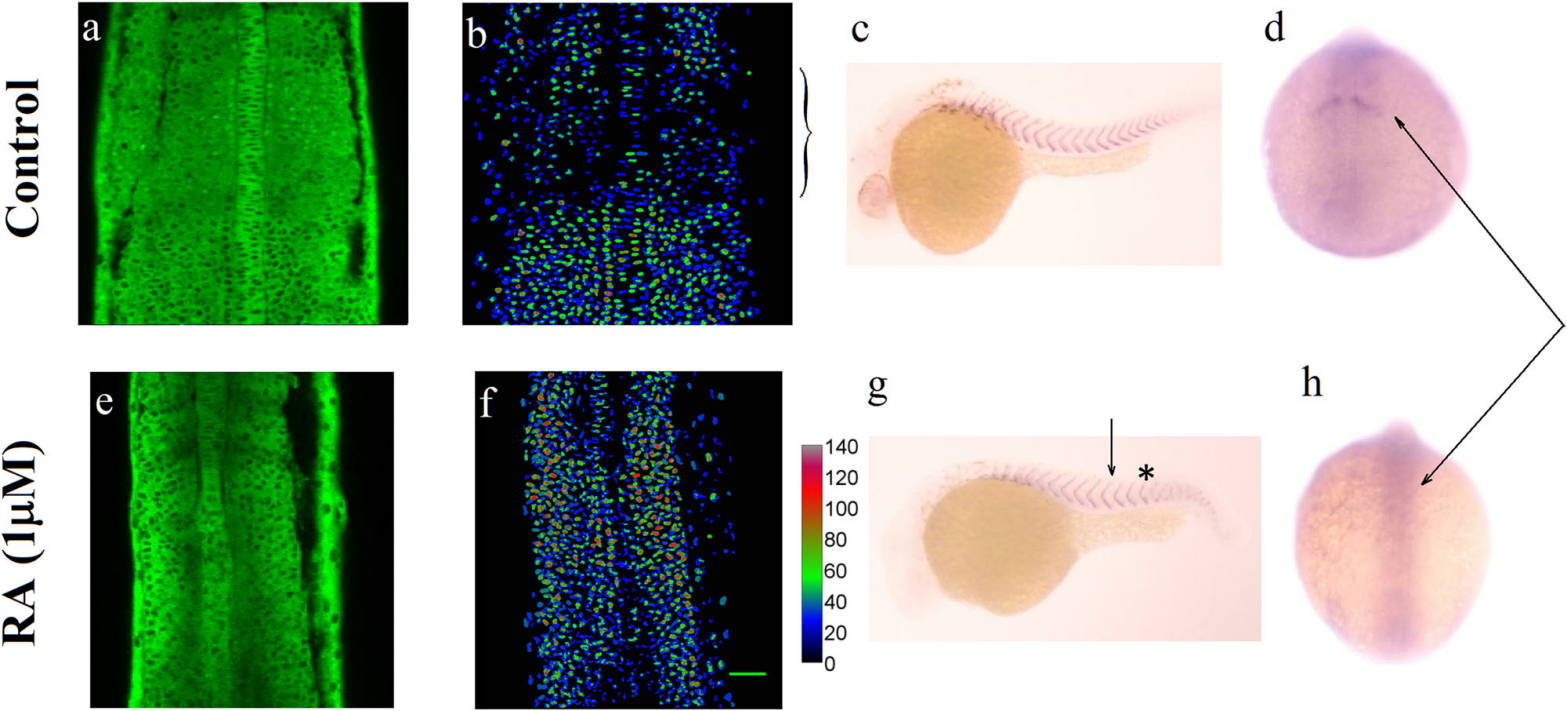
Somitogenesis at high Erk activity. **a**–**d** Control: normal development. **a** Erk activity in DREKA embryos at 14 somite stage: dark nuclei point to high Erk activity. **b** The false color images represent the Erk activity averaged over a 30 μm perpendicular stack of images. **c** ISH against *xirp2a* (a marker of somite boundaries) in 30 hpf embryos. **d** IHC against Mesp2a, a marker of the last somite boundary at 14 somites (arrow). **e**–**h** Somitogenetic development in DREKA embryos incubated from one-cell stage in 10 μM DEAB in presence of 1 μM RA from the ten somite stage (arrow in **g**). **e**, **f** The Erk activity is high throughout the PSM. **g** ISH against *xirp2a* in 30hpf embryos. Somitogenesis is impaired past 13 somites (indicated by a *) with unclear somite boundaries. **h** IHC against Mesp2a show that it is not expressed past 13 somite stage (arrows in **d** and **h**). Scale bar: 50 μm.

Next, we examined the role of RA in somitogenesis. Embryos were incubated from one cell stage in 10 μM DEAB (an inhibitor of RA synthesis) and exposed or not at 70% epiboly to 10 nM transRA (a physiological level which has been shown to rescue rhombomere formation following DEAB treatment^19^). These perturbations had minimal effects on the domain of activity of Erk (Fig. S2a) and on the dynamics of the somitogenetic wavefront (Fig. S2b). On the other hand, embryos treated with DEAB from the one-cell stage and incubated in 1 μM transRA at the onset of somitogenesis display a strong uniform activation of Fgf8 in the PSM^20^ (Fig. S3). Subjecting DREKA embryos to such a high RA concentration from the ten somite stage onward has dramatic effects: the Erk activity is uniformly enhanced throughout the PSM (Fig. 3e, f), and somitogenesis is impaired from 13 somites stage with unclear somite boundaries and no expression of Mesp2a^1^ (Fig. 3g, h).

The data shown in Figs. 2 and 3 suggest that a spatial gradient of Erk activity (or Fgf8) is required for proper somite formation. To check whether a temporal variation of Erk activity could also alter somite formation, we next studied somitogenesis in presence of an increasing uniform concentration of Fgf8 from an exogenous source.

### Uniform activation of an exogenous Fgf8 source does not affect somitogenesis

**Fig. 4:**
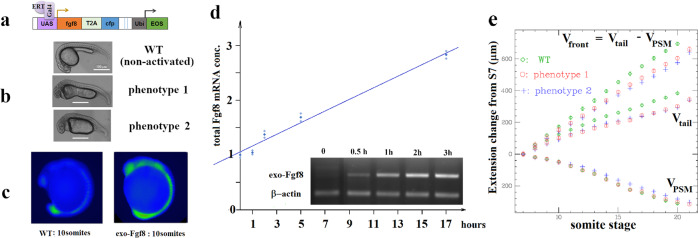
Somitogenesis upon induction of exogenous Fgf8. **a** The transgene *uas:fgf8-T2A-cfp* (under control of Gal4-ERT) and its marker Eos. **b** The two characteristic phenotypes observed at 24 hpf upon activation of the transgene. **c** Erk domain of activity visualized by IHC against phosphorylated Erk (pErk) at ten somites in non-activated embryos (left) or in embryos in which the transgene was activated from bud stage (right): the uniformly expressed exogenous Fgf8 enlarges the domain of activity of Erk to the whole embryo, but doesn’t alter the Erk activity gradient, Fig. S4. **d** Time variation of total *fgf8* mRNA (RTqPCR data) in presence of an exogenous source of Fgf8. At time *t* = 0 (bud stage) the concentration of *fgf8* is contributed by the endogenous one (continuous line: best linear fit *y* = 0.11 *x* + 1 with *r*^2^ = 0.98). From then on, the endogenous concentration decreases (see Fig. 5) while the exogenous concentration increases: it typically doubles the initial endogenous concentration in 9 h (about 20 somite stage). Inset: gel displaying the increase in exogenous *fgf8* mRNA at various times post activation versus a reference gene *(*β-*actin*). **e** Rates of PSM shrinkage (*V*_PSM_), tail growth (*V*_tail_), and wavefront velocity (*V*_front_) in embryos (*n* = 17) in which an exogenous source of Fgf8 was turned on. The various rates are only mildly affected by the increase in Fgf8 due to the exogenous source.

Our data in agreement with previous results^13^ suggest that somitogenesis is sensitive to the gradient of Fgf8 (or Erk activity) but insensitive to physiological perturbations of the RA concentration. To investigate the role of the Fgf8 variation during somitogenesis, we used a transgenic embryo *Tg*(*uas:fgf8-T2A-cfp*) to superpose an increasing but uniform source of Fgf8 on the endogenous one. The exogenous *fgf8* gene is turned on (Fig. 4a) when a transcription factor Gal4-ERT is released from its cytoplasmic chaperone complex by binding of cyclofen^21^.

Embryos uniformly expressing this exogenous source of Fgf8 display two characteristic phenotypes at 24 hpf, Fig. 4b: a mild phenotype (phenotype 1) characterized by enlarged heart and yolk and abnormal development of the head, and a severe phenotype (phenotype 2) characterized by enlarged heart, abnormal development of the head (with often no eyes), lost yolk extension and disordered late somites (>20 s). In contrast with embryos where the transgene has not been activated (similar to WT), upon expression of this exogenous source of Fgf8, Erk activity is observed throughout the embryo while still stronger at the posterior PSM (Fig. 4c). The continuous expression of the exogenous *fgf8* results in a total concentration of transcripts increasing roughly linearly with time (Fig. 4d), with a doubling of the initial (endogenous) concentration after about 8 h (20 somite stage). In spite of the presence of this uniformly expressed and continuously increasing exogenous source of Fgf8 on top of the endogenous one, the somitogenetic wavefront is barely affected and the PSM shrinks at the same rate as in WT embryos (Fig. 4e). In conjunction with the data shown in Figs. 2 and 3 this observation implies that the somitogenetic wavefront is sensitive to the spatial gradient of Fg8, not its local concentration, a conclusion reached earlier in different studies^13,18^.

### The endogenous Fgf8 concentration decreases during somitogenesis

If during somitogenesis the morphogen fields translate with the growing tail, namely if they are stationary in the tail moving frame, then one expects the PSM size to remain constant. Since the PSM shrinks one is forced to conclude that the morphogen fields are not stationary in the tail moving frame during somitogenesis: their amplitude and/or their extant^18^ may vary.

To investigate that question we decided to monitor by RTqPCR the mean endogenous *fgf8* mRNA concentration ($\left\langle \left[{{{fgf8m}}}\right]\right\rangle$ normalized by the mean concentration of *rpl13a*, a reference gene) during somitogenesis in the whole embryo and in the PSM. That concentration is related to the measured qPCR threshold cycle difference (*δCt*) via:

1 $$\delta {{{Ct}}}={{{\log }}}_{2}\left(\frac{\left\langle \left[{{{rpl13}}}\right]\right\rangle }{\left\langle \left[{{{fgf8m}}}\right]\right\rangle }\right) \sim -\frac{ {{{{{\mathrm{ln}}}}}}\left(\left\langle \left[{{{fgf8m}}}\right]\right\rangle \right)}{{{{{{\mathrm{ln}}}}}}2}$$

**Fig. 5:**
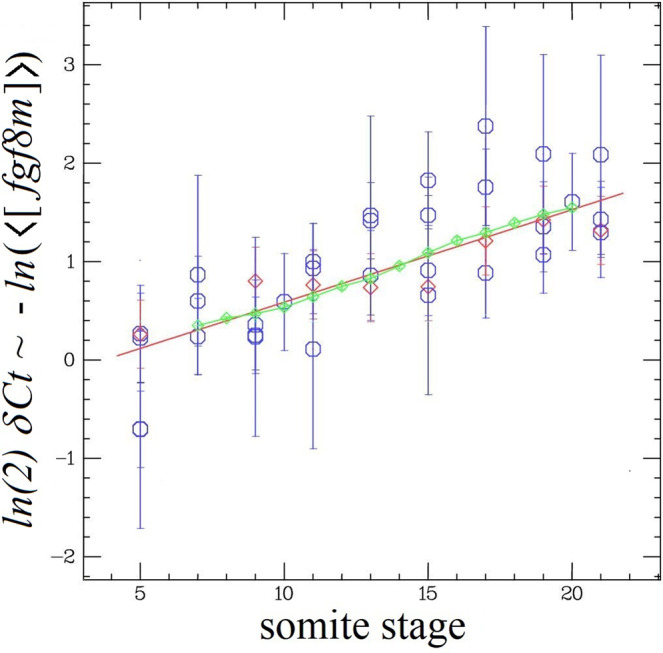
Variation of the fgf8 mRNA concentration with somite stage. Negative *ln*(<[*fgf8m*]>) (=ln(2) *δCt* values from the RTqPCR data) at different somitic stages (blue ○: whole embryo; red ◊ : PSM only). Red line: linear best fit (*y = ax* + *b*) with *a* = 0.094 ± 0.013 (*Χ*^2^ *=* 22; DF = 36), corresponding to a an exponential decrease of <[*fgf8m*]> with a time constant *τ* = 1/*a* ≅ 11 somitic periods, Eq. (1). Green ◊: -ΔPSM/λ with *λ* = 260 μm and the PSM shrinkage, ΔPSM, taken from Fig. 4e. From the value of λ and τ we deduce a mean velocity: *V*_PSM_ = *λ*/*τ* ≅ 24 μm/somite.

In both whole embryo and PSM, we observed an overall linear increase in the *δCt* values between the 5 and 21 somite stages, see Fig. 5, corresponding to an exponential decrease in the mean fgf8 concentration $\left\langle \left[{{{fgf8m}}}\right]\right\rangle \sim {{\exp }}(-t/\tau )$ with a typical timescale: *τ* ~4.5 h or about 11 segmentation periods.

In that time interval the PSM shrinks at a constant rate^5^ (see Fig. 4e): ΔPSM = *V*_PSM_ ∙ t, with *V*_PSM_ ~58 μm/h = 24 μm/somite at 27 °C. Therefore, one can also relate the decrease in $\left\langle \left[{fgf}8m\right]\right\rangle$ to the PSM shrinkage: $\left\langle \left[{fgf}8m\right]\right\rangle \sim {{\exp }}(-\frac{\Delta {{{{{\rm{PSM}}}}}}}{{{{{{\rm{\lambda }}}}}}})$, where: λ = τ V_PSM_ ~ 260 μm is a characteristic length roughly equal to the extent of diffusion of the Ffg8 morphogen field^22^.

### The dynamics of *fgf8* slows down with decreasing temperature by the same factor as the PSM shrinkage rate

**Fig. 6:**
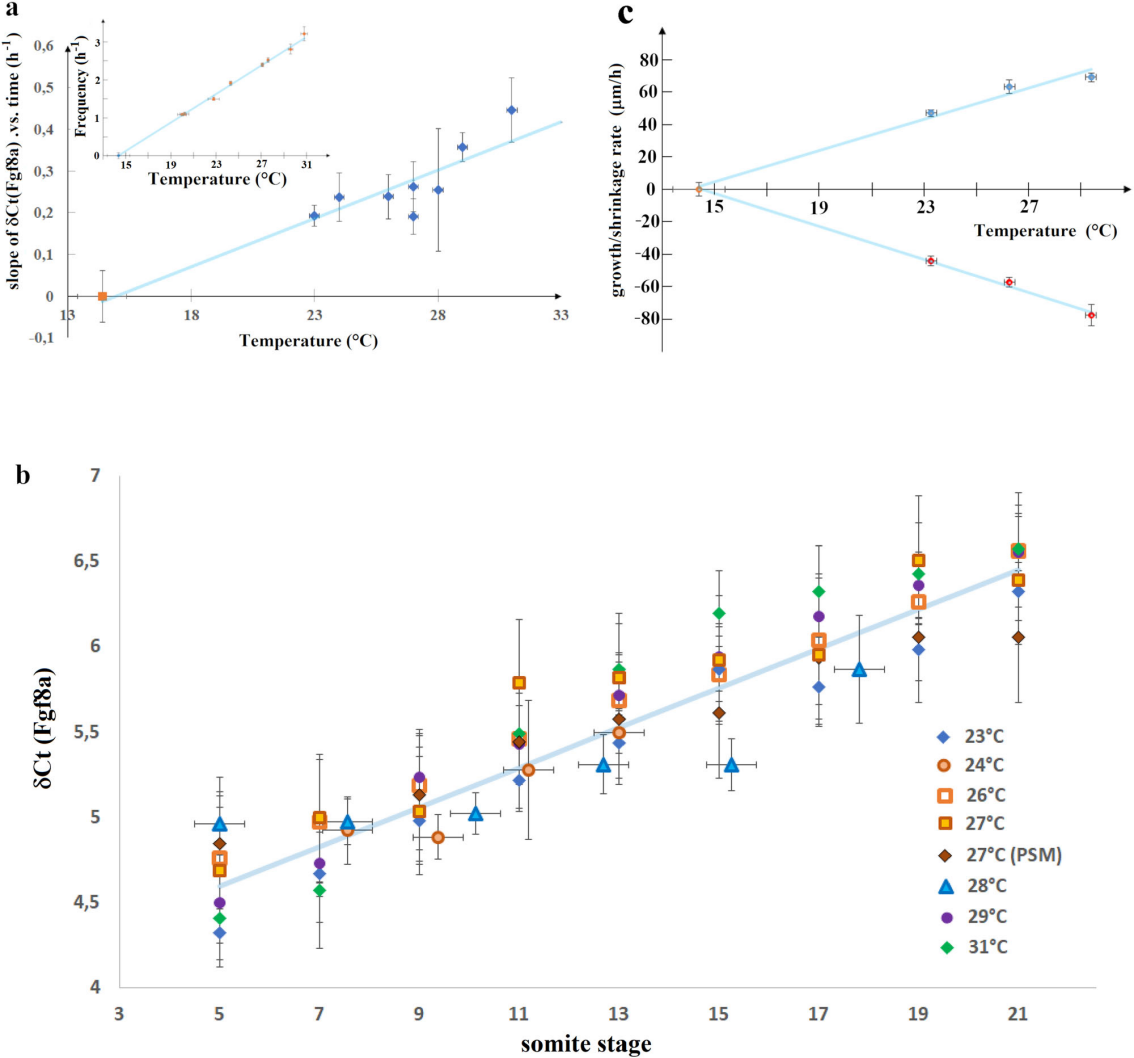
Variation with temperature of the dynamics of fgf8 mRNA, tail growth and PSM shrinkage. **a** Variation with temperature of the slope α of *δCt*(*fgf*8(*t*)) vs time (Fig. 5), which is linearly related to the exponential decay rate of <[*fgf8m*]>: 1/*τ* = ln(2) *α*. The red point at 27 °C is from PSM only data. The data can be fit (blue line) by *α* = *a* (*T*−*T*c) with: *a* = 0.022 ± 0.0038; *T*_c_ = 14.4 °C (*Χ*^2^ = 6.57; DF = 6). Inset*:* variation of the clock frequency *f*_s_ = *1/τ*_s_ vs temperature^2^ (data from Schröter et al.^2^) which can be fit by *f*_s_ *=* a_*s*_ (*T* − *Tc*) with *a*_*s*_ = 0.188. **b** Another w*a*y to represent the data shown in **a**: since *δCt*(*fgf*8 (*t*)) varies with temperature in a similar way as the segmentation period, *τ*_s_, the δ*Ct*(*fgf*8(*t*)) data taken at different temperatures collapse on the same curve as a function of somite stage: *s* = *t/τ*_s_ (=number of segmentation periods). The data at all temperatures can be fit (blue line) by *δCt = a*′ *s* + *b*, with *a*′ = 0.122 ± 0.0098 and *b* = 3.955 (χ^2^ = 26.5; DF = 55). Notice that as expected: *a* = *a*′*∙ a*_s_. **c** variation with temperature of the growth rate (blue points) and the PSM shrinkage rate (red points) and linear best fits (blue lines): *y* = *β*(*T* − Tc) with *β* = 4.79 ± 0.46 (χ^2^ = 6.35; DF = 2; blue points) and *β* = −5.08 ± 0.20 (χ^2^ = 0.51; DF = 2; red points).

The data obtained previously at 27 °C suggest that the PSM shrinkage rate *V*_PSM_ = *λ*/*τ* is inversely proportional to the characteristic decay time *τ* of the mean *fgf8* mRNA concentration. Since *V*_PSM_ varies significantly with the temperature^2^ (see Fig. 6c), if that relation is not fortuitous it should hold at all temperatures, namely the variation of 1/*τ* with temperature should be the same as that of *V*_PSM_. To investigate that point we studied the time dependence of the mean *fgf8* mRNA concentration at various temperatures.

At all temperatures we observed that the *fgf8* *δCt* values (between 5 and 21 somites) increase linearly with time, i.e., $\left\langle \left[{fgf}8m\right]\right\rangle$ decays exponentially with time. The typical timescale *τ* of that exponential decay (i.e., the slope of *δCt* vs. time, see Eq.(1)) varies with temperature as: 1/*τ* ≅ 0.0152 (*T* − *T*_c_) h^−1^ with *T*_c_ = 14.4 °C (Fig. 6a, b), a behavior similar to the variation with temperature of the growth rate and PSM shrinkage rate, see Fig. 6c. Therefore, the relation between the PSM shrinkage and the *fgf8* decay rate holds at all temperatures and is not fortuitous. Notice also that the divergence of *τ* with temperature is similar to that of the segmentation period^2^: *τ*_s_ ≅ 5.32/(*T* − *T*_c_) hours (see inset in Fig. 6a).

**Fig. 7:**
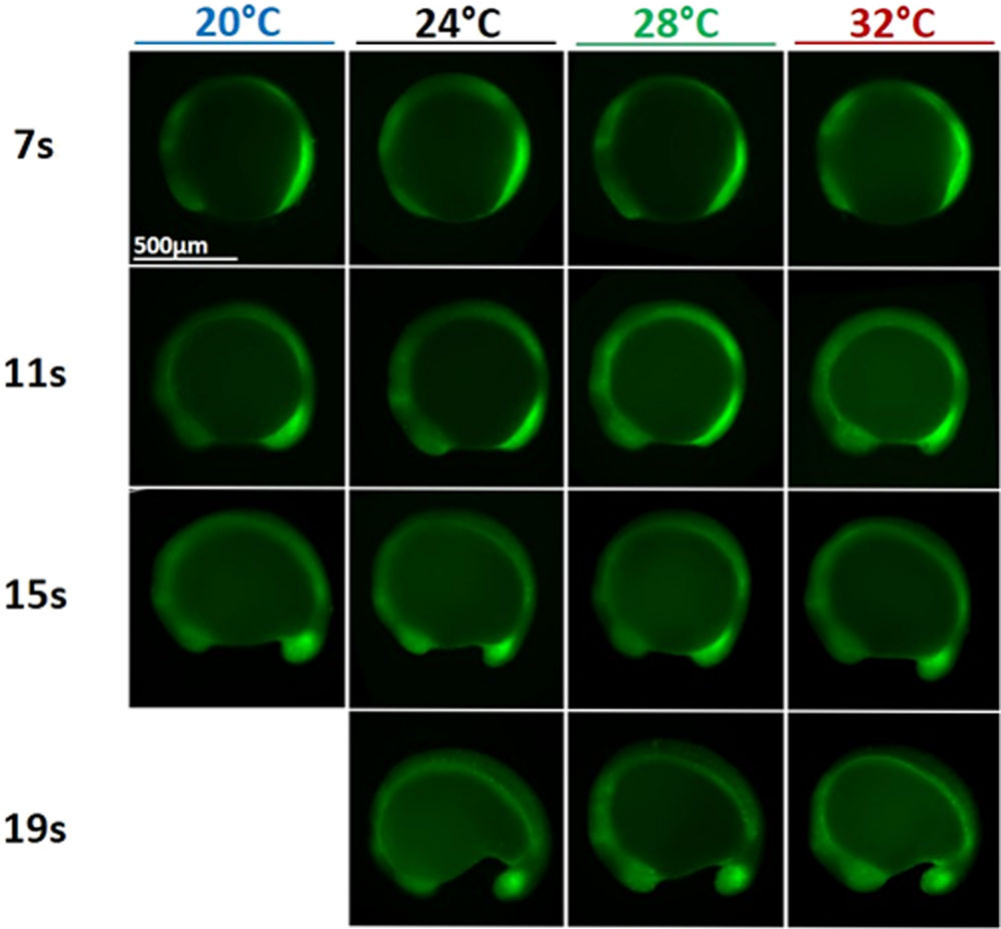
Somitogenesis at different temperatures. Phosphorylated Erk (pErk) distribution in WT embryos grown at different temperatures (20, 24, 28, and 32 °C) at 4 different stages of somitogenesis (7, 11, 15, or 19 somites stages) visualized by fluorescence following Immunohistochemistry staining (IHC). Even though embryos at 20 °C grow about three time slower than embryos at 32 °C, at similar stages embryos grown at different temperatures are similar and display similar pErk domain.

These observations on the temperature independence of the average level of *fgf8* mRNA at given stages of somitogenesis (Fig. 6b) are further supported by measurements of the spatial activity of Erk, a downstream effector of Fgf8. As shown in Figs. 7 and S5, the spatial distribution and intensity of pErk is temperature independent: it is the same at similar stages of somitogenesis but different temperatures (from 20 to 32 °C).

### The dynamics of many gene transcripts active during somitogenesis slow down with temperature as *T* − *T*_c_

The variation with temperature of the segmentation period, tail growth rate, PSM shrinkage rate and *fgf8* decay rate are characteristic of the critical slowing down of a system near *T*_c_. If somitogenesis is indeed a critical system, all time-varying gene transcripts should behave similarly, i.e., their characteristic time should scale as 1/(*T* − *T*_c_) or stated differently their variation as a function of the somite stage, *s* = *t/τ*_s_ should be temperature independent. To address this question, we studied, using RTqPCR, the dynamics of the relative average concentration of many gene transcripts at different temperatures, see Table S1. If they scale with temperature in the same way as the segmentation clock period, the data for a given transcript taken at different temperatures should fall on the same curve.

**Fig. 8:**
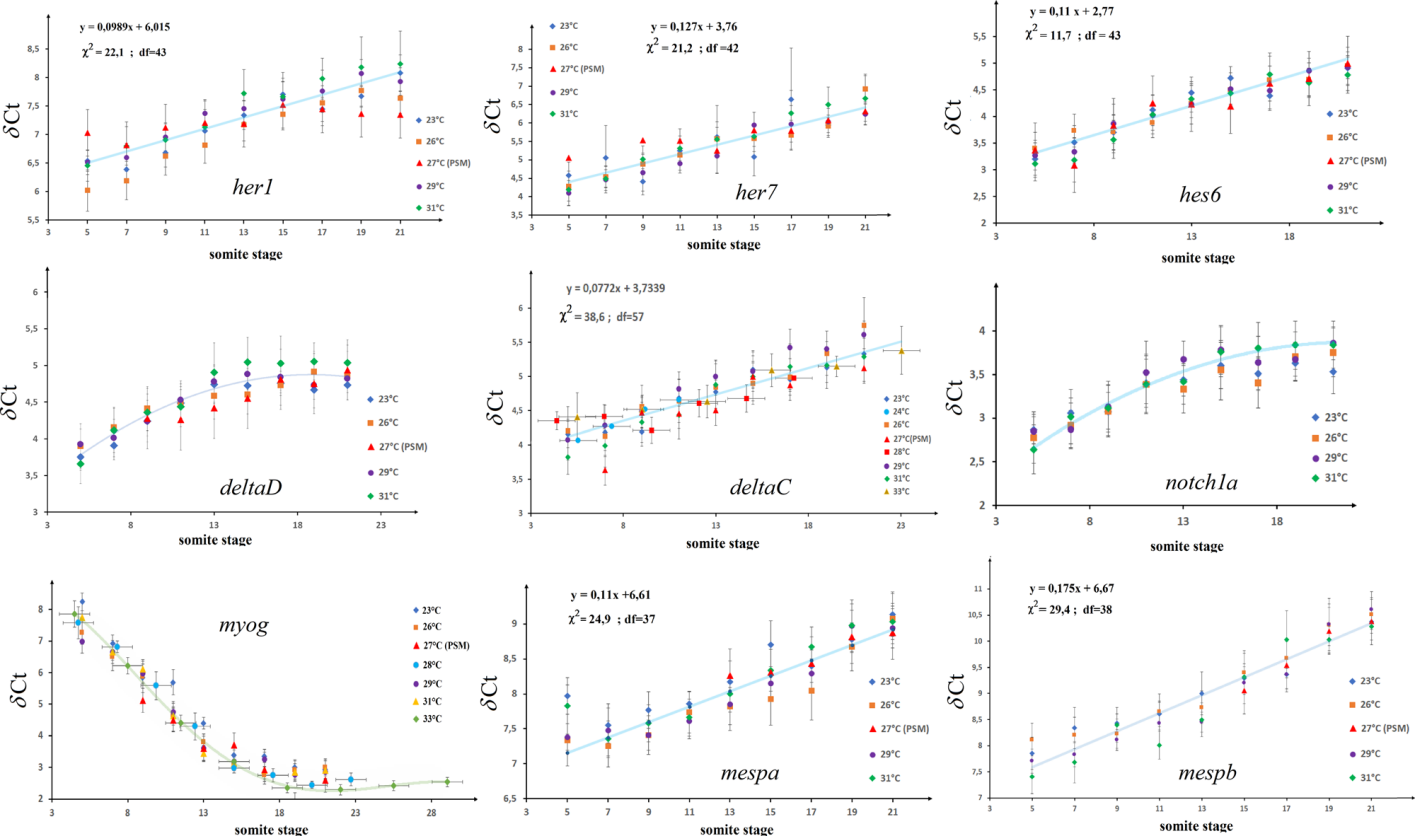
Variation with temperature of various gene transcripts. The *δCt* of genes (*her1, her7, hes6, deltaC, deltaD, notch1a, myog, mespa*, and *mespb*) known to play a role in somitogenesis relative to reference genes (*rpl13a* or β*-actin*) are plotted as a function of somite stage. Notice that as a function of somite stage the data taken at different temperatures collapse on the same curve. For some genes for which *δCt* appears to vary linearly with somite stage (exponentially decreasing concentrations) we display the best fit. For others the continuous curve is just a guide to the eye. The increasing concentration of *myog* (decreasing *δCt* values) is consistent with its role as a differentiation factor during myogenesis which follows somite formation.

This is the case for genes known to play a role during somitogenesis^23^, see Fig. 8, such as the genes implicated in the segmentation clock^24^ (*her1, her7,* and *hes6*) and its synchronization^25^ (*deltaC, deltaD,* and *notch1a*), in segmental patterning (*mespa* and *mespb*) and myogenesis (*myog*) but also for genes involved more generally in cellular differentiation (*vox, ventx, nanog*, and *oct4*) (Fig. S6). For genes which *δCt* value vary linearly with time during somitogenesis we can compute the slope of the linear best fit at various temperatures (Figs. 6a and  S7). This analysis confirms the critical slowing down of genes as the temperature is decreased. Notice however that not all genes vary during somitogenesis. For some of them (*fgf4, igf2a, wnt3,* and *xpc)*, the relative mean concentration seems constant between 5 and 21 somite stage (Fig. S8).

## Discussion

In this work, we have studied some factors affecting the somitogenetic wavefront velocity, in particular the PSM shortening rate. The observed impairment of somitogenesis in absence of an Erk activity gradient (with either uniformly high or low Erk phosphorylation) suggests, in agreement with recent reports^13,18^, that it is the spatial variation of Fgf8 (via its downstream effector, Erk) and not its local concentration which sets the position of the somitogenetic wavefront. This conclusion is further confirmed by the observed invariance of somitogenesis in presence of a uniformly increasing concentration of Fgf8. The observed periodic jumps in the anterior boundary of the Erk activity domain moreover imply that commitment to somite formation is triggered when the gradient of Fgf8 falls below a threshold at a certain phase of the segmentation clock.

The shortening of the PSM therefore implies that the Fgf8 gradient decays with time. To investigate that issue we have measured the mean *fgf8* mRNA concentration during somitogenesis (in whole embryo and in the PSM). Our observations point to an exponential decrease of the mean *fgf8* mRNA concentration with a typical time scale of *τ* ~11 somitogenetic periods: $\left\langle \left[{fgf}8m\right]\right\rangle \sim {{\exp }}(-t/\tau )$. Since the PSM shrinks at a constant rate *V*_PSM_ ~24 μm/somite: ΔPSM = *V*_PSM_ *t*, the mean *fgf8* mRNA decay can also be expressed as a function of the PSM size: $\left\langle \left[{fgf}8m\right]\right\rangle \sim {{\exp }}(-\frac{\Delta {{{{{\rm{PSM}}}}}}}{{{{{{\rm{\lambda }}}}}}})$, with *λ* = *τ* *V*_PSM_ ~ 260 μm.

These results are consistent with a source-sink mechanism for the Fgf8 gradient^22^, where the *fgf8* mRNA is generated in the progenitor domain with a time decreasing concentration (see Supplementary Note 1) and is degraded on a typical length-scale *λ* towards the anterior part of the PSM: [*fgf8m*(*x,t*)] = *F*_0_ exp(−*x/λ* – *t/τ*) ≡ *u(x,t)*, where *x* is the distance to the tail end. Thus, the measured mean concentration $\left\langle \left[{fgf}8m\right]\right\rangle$ varies exponentially with time, as indeed observed:

2 $$\left\langle \left[{fgf}8m\right]\right\rangle ={\int }_{0}^{{{\infty }}}{{{d}}x}\left[{fgf}8m(x,t)\right] \sim {{\exp }}(-t/\tau )$$

Our results suggest that a new somite *n* is formed at distance *x*_n_ from the tail end when the gradient of Fgf8(*x,t*) falls below a certain threshold, *η*_t_ at a given phase of the somitogenesis clock*: t* = *nτ*_s_.

3 $${\eta }_{{{{{{\mathrm{t}}}}}}}=-\frac{{{{{{{\mathrm{d}}}}}}Fgf}8}{{{{{{{\mathrm{d}}}}}}x}}\left({x}_{{{{{{\mathrm{n}}}}}}},n{\tau }_{{{{{{\mathrm{s}}}}}}}\right)$$

If the concentration of Fgf8 protein is a monotonous function of its mRNA: Fgf8(*x,t*) = G(*fgf8m*(*x,t*)), then:

4 $${\eta }_{{{{{{\mathrm{t}}}}}}}=\frac{1}{\lambda }{u}_{{{{{{\mathrm{t}}}}}}}{G}_{{{{{{\mathrm{u}}}}}}}^{{\prime} }\left({u}_{{{{{{\mathrm{t}}}}}}}\right)=\frac{{F}_{0}}{\lambda }{{\exp }}\left(-\frac{{x}_{{{{{{\mathrm{n}}}}}}}}{\lambda }-\frac{n{\tau }_{{{{{{\mathrm{s}}}}}}}}{\tau }\right){G}_{{{{{{\mathrm{u}}}}}}}^{{\prime} }\left({u}_{{{{{{\mathrm{t}}}}}}}\right)$$

In other words: ${x}_{{{{{{\mathrm{n}}}}}}}={x}_{0}-{n\tau }_{{{{{{\mathrm{s}}}}}}}\lambda /\tau ={x}_{0}-n{\tau }_{{{{{{\mathrm{s}}}}}}}{V}_{{{{{{{\mathrm{PSM}}}}}}}}$ with ${x}_{0}=\lambda {{{{{\rm{ln}}}}}}({G}_{{{{{{\mathrm{u}}}}}}}^{{\prime} }({u}_{{{{{{\mathrm{t}}}}}}}){F}_{0}/{\eta }_{{{{{{\mathrm{t}}}}}}}\lambda )$. Namely the PSM shrinks at a velocity entirely determined by the dynamics of Fgf8 (the ratio between its degradation length-scale *λ* and its decay rate *τ*) (Fig. S9 and Supplementary Note 1). Notice that if the threshold (i.e., *x*_0_) is altered by a drug such as SU5402 from somite stage *n* onwards, only the size of the first somite after the perturbation *l*_n+1_ = *x*_n+1_ − *x*_n_ will be altered (as *x*_n+1_ and *x*_n_ are associated to different thresholds (i.e., *x*_0_)), while later somites will have a regular size (same threshold, i.e., *x*_0_), as indeed observed^18^. It may be interesting to check in other model organisms if $\left\langle \left[{fgf}8m\right]\right\rangle$ decays exponentially with time and if differentiation into somite is set by its gradient. If so one expects the *fgf8* mRNA concentration to correlate with the PSM size as observed here, i.e., as exp(−ΔPSM/λ).

Our results are also compatible with a recent report of a scaling relation between somite and PSM size in tail explants^13^, if the time dependence of the *fgf8* mRNA concentration in these tail explants does not decay exponentially (Fig. S9 and Supplementary Note 1).

We have further observed that the relation between the *fgf8* decay time, *τ*, and the PSM shrinkage rate *V*_PSM_: *V*_PSM_ = *λ*/*τ* was conserved over a temperature range, where *V*_PSM_ varies by a factor two, implying that this relation established initially at 27 °C is not fortuitous. We observed that the *fgf8* decay rate, the spatio-temporal behavior of its downstream effector (pErk), the PSM shrinkage and tail growth rates and the clock frequency, all display the characteristic behavior of a system near its critical temperature at *T*_c_ = 14.4 °C: all rates slow down as *T* − *T*_c_. We have generalized that observation to many time-varying genes implicated in somitogenesis and cellular differentiation. These results imply that somitogenesis shares the characteristic dynamics of a system near criticality^26^ (all times-scales increase as 1/(*T* − *T*_c_), see Supplementary Note 1), even though the critical temperature is experimentally not achievable (embryos die below about 19 °C). Our observations may explain the invariance of the developmental process to temperature even if its characteristic times-scale changes by a factor 2–3. In other words, there is no need for a specific thermal compensation mechanism to keep all the relevant genes in synchrony, further highlighting the robustness of that system.

One may speculate about the biological significance of the critical temperature. Its value is close to the lowest recorded temperature of the natural environment of zebrafish^27^. It is also close to the critical temperature below, which swimming is impaired^28^ and lethality in adult fish increases^27^. This suggests that zebrafish evolution has tuned some metabolic networks to stop functioning below Tc. In certain fish^29^, a mechanism known as diapause, can be triggered at certain developmental stages (e.g., mid-somitogenesis) and under certain environmental conditions (such as too high or too low temperatures), which cause the developing animal to enter a state of dormancy. A diapause-like state has been reported in zebrafish, though induced by blockage of the vitamin D synthesis pathway, not by exposure to a low temperature^29^. It is tempting to speculate that a critical mechanism that causes metabolic networks to stop working below a given temperature and is lethal in zebrafish could have been coopted in other species to shut down some networks and enter a dormancy state.

## Methods

### Fish lines and maintenance

Zebrafish were raised and maintained on a 14–10 h light-dark diurnal cycle with standard culture methods^30^. Embryos collected from natural crosses were staged according to Kimmel^31^. The *Tg(uas:fgf8-T2A-cfp;ubi:eos)* was generated by injecting the plasmid *pT24-uas:fgf8-T2A-cfp;ubi:Eos* (a gift from M. Volovitch), which contains the homologous cDNA sequence of *fgf8* from Danio rerio, with *tol2* mRNA transposase. Founder transgenic fish were identified by global expression of Eos. The *Tg*(*ubi:gal4-ERT;cry:CFP)* was previously described^32^. The double transgenic line *Tg(ubi:gal4ERT;cry:CFP; uas:fgf8-T2A-cfp;ubi:Eos)* was created by crossing *Tg(uas:fgf8-T2A-cfp;ubi:eos)* and *Tg*(*ubi:gal4-ERT;cry:CFP)*. Founder double transgenic fish were selected by global expression of Eos and expression of CFP in the developing eyes. The transgenic line *tg(ubi:ERK-KTR-Clover)*^*vi001*^ (DREKA) allowing for real time monitoring of Erk activity in live embryos has been described elsewhere^15^. Embryos were imaged for phenotypic analysis at 24 hpf and then fixed with PAXgene Tissue Container Product (Qiagen) for RT-PCR.

### RT-PCR

Extraction of total RNA was performed by using RNeasy Micro Kit (Qiagen), according to the manufacturer’s instructions. cDNA synthesis was performed using sensiscript reverse transcriptase (Qiagen) with an anchored Oligo(dT)23VN primer (NEB). PCR was performed using the Phusion High-Fidelity DNA Polymerase (NEB) with the following protocol: 98 °C for 30 s; then 30 cycles (98 °C for 10 s, 60 °C for 30 s, 72 °C for 30 s); 72 °C for 10 min. A pair of primers, P1 (5′-ACCATTCAGTCCCCGCCTAA-3′) and P3(5′-GCCAATCAGTTTCCCCCTCC-3′), which respectively match exon3 and exon4 of Fgf8, were used to detect the expression of the pre-mRNA of the endogenous Fgf8 resulting in a 1900 bp PCR product and the expression of the exogenous and correctly spliced Fgf8 resulting in a 283 bp PCR product. Another pair of primers, P3 (same as before) and P2 (5′-CCCCTCCGTTTGAACCGTAA-3′) against intron3 of Fgf8, was used to analyze the expression of the pre-mRNA of the endogenous Fgf8 with a PCR product of 722 bp. This pair cannot amplify the exogenous and correctly spliced Fgf8. Actb2 was amplified as a reference gene with the following primers: 5′-TGTACCCTGGCATTGCTGAC-3′ and 5′-CCATGCCAATGTTGTCGTTTG-3′.

### Reverse transcriptase quantitative PCR (RT-qPCR)

Wild type embryos (10–15 embryos in each group) grown in an incubator at a given temperature (23, 26, 27, 29, or 31 °C) were fixed (as mentioned above) at the somite stages mentioned in the text. In some experiments (24, 28, and 33 °C) embryos were fixed at intervals of 1 h after onset of somitogenesis, though this procedure resulted in less reproducible and consistent results, possibly due to differences in developmental stage between the embryos. For gene quantification on specific tissue, single PSM (15 in each group) were dissected from fixed WT embryos at the last somite boundary.

#### RNA extraction

Total RNAs were extracted using the RNeasy micro kit (Qiagen) according to the manufacturer’s protocols. Contaminating genomic DNA was degraded by treatment with DNaseI (Qiagen) for 20 min at room temperature. Total RNAs were eluted in 14 µL nuclease-free water (Qiagen). RNA concentration and purity were first assessed using NanoDrop (ThermoFisher). Sample quantity and purity were estimated by measuring the ratios of spectrophotometric absorbance 280/260 nm and 260/230 nm. RNA quality and integrity were further analyzed by capillary electrophoresis (Fragment Analyzer, Agilent Technologies) to determine the RNA quality number (RQN) for each sample. Defined on a scale ranging from 1 to 10, the mean RQN of the 194 samples was 9.9, indicating very good RNA quality. RNA were stored at −80 °C before reverse transcription.

#### Reverse transcription

RNA samples were diluted at 10 ng/µL by adding nuclease-free water. cDNA synthesis was performed using Reverse Transcription Master Mix from Fluidigm™ according to the manufacturer’s protocol in a final volume of 5 μL containing 40 ng total RNA. Reverse transcription was performed using a Nexus Thermocycleur (Eppendorf^®^) following the temperature protocol: 5 min at 25 °C, 30 min at 42 °C followed by heat-inactivation of the reverse transcriptase for 5 min at 85 °C and immediately cooled to 4 °C. cDNA samples were diluted 5× by adding 20 μL of low TE buffer [10 mM Tris; 0.1 mM EDTA; pH = 8.0] (TEKNOVA^®^) and stored at −20 °C before specific target pre-amplification.

#### Specific target pre-amplification

Each diluted cDNA was used for multiplex pre-amplification in a total volume of 5 μL containing 1 μL of 5× Fluidigm^®^ PreAmp Master Mix, 1.25 μL of cDNA, 1.25 μL of pooled TaqMan^®^ Gene Expression assays (Life Technologies, ThermoFisher) with a final concentration of each assay of 180 nM (0.2×) and 1.5 μL of nuclease-free water. The cDNA samples were subjected to pre-amplification following the temperature protocol: 95 °C for 2 min, followed by 18 cycles at 95 °C for 15 s and 60 °C for 4 min. The pre-amplified cDNA was diluted 5× by adding 20 μL of low TE buffer and stored at −20 °C before qPCR.

#### High throughput qPCR

Quantitative PCR was performed using the high-throughput platform BioMark™ HD System and the 48.48 GE Dynamic Arrays (Fluidigm^®^). The expression of 48 target genes was quantified in 108 samples by quantitative PCR on five 48.48 microfluidic chips. Each of the five chips contained a non-template control (NTC), and a serial dilution of a cDNA sample has been used both as a standard curve to determine efficiencies and inter-chip calibrator. The Ct obtained from the standard curve and an internal control were identical among the five chips allowing inter-chip comparisons. Six microliter of Sample Master Mix (SMM) consisted of 2.7 μL of 5× diluted pre-amplified cDNA, 0.3 μL of 20× GE Sample Loading Reagent (Fluidigm) and 3 μL of TaqMan^®^ Gene Expression PCR Master Mix (Life Technologies, ThermoFisher). Each 6 μL Master Mix Assay (MMA) consisted of 3 μL of TaqMan^®^ Gene Expression assay 20× (Life Technologies, ThermoFisher) and 3 μL of 2× Assay Loading Reagent (Fluidigm). Five microliter of each SMM and each MMA premixes were added to the dedicated wells. The samples and assays were mixed inside the chip using MX IFC controller (Fluidigm). The loaded Dynamic Array was transferred to the Biomark™ real-time PCR instrument and subjected to PCR experiment (25 °C for 30 min and 70 °C for 60 min for thermal mix; 50 °C for 2 min and 95 °C for 10 min for hot start; 40 cycles at 95 °C for 15 s and 60 °C for 1 min). The parameters of the thermocycler were set with ROX as passive reference and single probe FAM-MGB as fluorescent detector. To determine the threshold cycle Ct, data were processed by automatic threshold for each assay, with linear derivative baseline correction using BioMark Real-Time PCR Analysis Software 4.0.1 (Fluidigm). The quality threshold was set at the default setting of 0.65.

#### Normalization and quantification

The reference gene *rpl13a* (or β*-actin* when mentioned in the text) has been chosen after pair-wised correlation analysis using Bestkeeper algorithm^33^. The difference in the number of quantification cycles between the genes of interest (GOI) (in various conditions) and a reference gene (GRF, i.e., *rpl13a or* β*-actin*) was *δ*Ct = Ct_GOI_ – Ct_GRF_. The normalized transcripts abundance (averaged over three PCR replicates) was calculated as $\delta {{{{{{\mathrm{Ct}}}}}}}={{{{{{\mathrm{log }}}}}}}_{2}\left(\left\langle \left[{rpl}13a\right]\right\rangle /\left\langle \left[{{{{{{\mathrm{goi}}}}}}}\right]\right\rangle \right) \sim -{{{{{\mathrm{ln}}}}}}\left(\left\langle \left[{{{{{{\mathrm{goi}}}}}}}\right]\right\rangle \right)/{{{{{\mathrm{ln}}}}}}2$. The list of genes studied is described in Table S1.

### IHC on zebrafish embryos

The embryos obtained in the various conditions and the different stages mentioned in the text were fixed in 4% PFA overnight at 4 °C, followed by dehydration with 100% methanol at −20 °C for more than 1 day. After gradual rehydration of methanol and wash with PBS/Tween 0.1%, the embryos were incubated in a blocking solution: 1%Triton, 1% DMSO, 1% BSA and 10% sheep serum (Sigma) in PBS on a shaker for 1 h at room temperature, followed by incubation with 1:400 Phospho-ERK Monoclonal Antibody as the primary antibody (Thermo, MA5-15173) on a shaker overnight at 4 °C. After extensive washing with PBS/Tween and incubation in blocking solution, a second antibody (anti-Rabbit conjugated to Alexa Fluor 488, Invitrogen) diluted 700× was added overnight at 4 °C. After washing with PBS/Tween, images were taken on a Nikon Ti microscope equipped with a Hamamatsu Orca camera.

### Whole-mount in-situ hybridization (ISH)

Whole-mount in-situ hybridization with digoxigenin-labeled riboprobes was performed as described previously^34^. The antisense riboprobes were synthesized from template plasmids (gift of P. Rosa, IBENS) containing fgf8 full length cDNA^35^. The antisense riboprobes were synthesized from template plasmids containing fgf8 full length cDNA^34^ or xirp2a^36^.

### Drug treatments

Wild type embryos were incubated in 10 μM DEAB (with or without addition at 70% epiboly of 10 nM all-trans RA). The double transgenic embryos *Tg(ubi:Gal4ERT;cry:CFP;uas:fgf8-T2A-cfp;ubi:Eos)* were incubated at 70% epiboly in 3 μM cyclofen^37^ (gift of L. Jullien) diluted in EM. DREKA embryos were treated with 50 μM, 200 μM of SU5402 (Sigma-Aldrich) at 10 somite stage for 1 h and 10 nM and 1 μM of all-trans RA (Sigma-Aldrich) from ten somite stage until observation. As controls, siblings were kept in EM.

### Fluorescent microscopy

DREKA embryos were imaged on a Leica sp5 confocal microscope with a 20× water objective in GFP channel. All other fluorescent images were taken on a Nikon Ti microscope equipped with a Hamamatsu ORCA V2+ camera and a 10× plan fluo objective. Filter setting of CFP: excitation at 438 ± 24 nm, emission at 483 ± 32 nm; mEosFP and Alexa 488: excitation at 497 ± 16 nm, emission at 535 ± 22 nm.

### Time lapse video

All embryos were dechorinated before bud stage using acute tweezers. The embryos were mounted in an agarose mold and kept in a temperature incubator at 27 ± 0.3 °C during imaging, following published protocols^38^. To prevent evaporation during recording caused by the difference in temperature between the microscope room and the incubator, mineral oil (Sigma) was added to cover the surface of the medium. Time-lapse video was performed on a Nikon Ti Microscope with a 10× plan fluo objective. Images were taken by an ORCA V2 + Camera (Hamamatsu). The entire microscope system including the filter rotor, the motorized stage and the camera was driven by Micro-Manager. Up to 20 embryos per experiment were scanned at an interval of ~5 min. For each embryo, a Z-stack with six planes at 25 μm intervals was recorded.

### Image analysis of time lapse video

Time lapse images were analyzed by FIJI. The Gaussian-based stack focuser in FIJI Time Lapse plugin was used to choose and combine the focused areas from different Z-positions of one timepoint. The measurement of tail, PSM and somite length was done using the measurement function in FIJI. ERK activity of DREKA was quantified in FIJI by the ratio between the mean fluorescence of nucleus and a 2-pixel wide cytoplasm ring.

### Statistics and reproducibility

To assess the reproducibility of our observations, we repeated them on many embryos. Thus, measurements of growth rates and PSM shrinkage rates in different conditions were obtained from experiments on typically *n* > 10 embryos (see captions to Fig. 4e and S2b). RTqPCR measurements were done in triplicate on pooled embryos and repeated in full embryos or in the PSM (at 27 °C). Similarly experiments on the domain of activity of Erk, were done over many (*n* > 16) embryos at 27 °C (Fig. S4) and over *n* = 3 embryos at different stages and temperatures (Fig. S5). Linear fits to the data were done with maximum likelihood estimates: the value of *χ*^2^ and the number of degrees of freedom (*df*) are indicated next to each fit (Figs. 7, S6, and S7) or in the figure captions (Figs. 5 and 6).

### Reporting summary

Further information on research design is available in the Nature Research Reporting Summary linked to this article.

## Supplementary information

Transparent Peer Review File

Supplementary Information

Description of Additional Supplementary Files

Supplementary Data 1

Reporting Summary

## Data Availability

Raw data are provided in Supplementary Data 1 or are available upon request from the authors.

## References

[1] Stickney HL, Barresi MJF, Devoto SH (2000). Somite development in zebrafish. Dev. Dyn..

[2] Schröter C (2008). Dynamics of zebrafish somitogenesis. Dev. Dyn..

[3] Cooke J, Zeeman EC (1976). A clock and wavefront model for control of the number of repeated structures during animal morphogenesis. J. Theor. Biol..

[4] Dequéant M-L, Pourquié O (2008). Segmental patterning of the vertebrate embryonic axis. Nat. Rev. Genet..

[5] Bajard L (2014). Wnt-regulated dynamics of positional information in zebrafish somitogenesis. Development.

[6] Soroldoni D (2014). Genetic oscillations. A Doppler effect in embryonic pattern formation. Science.

[7] Gomez C (2008). Control of segment number in vertebrate embryos. Nature.

[8] Dubrulle J, McGrew MJ, Pourquié O (2001). FGF signaling controls somite boundary position and regulates segmentation clock control of spatiotemporal Hox gene activation. Cell.

[9] Sawada A (2001). Fgf/MAPK signalling is a crucial positional cue in somite boundary formation. Development.

[10] Delfini M-C, Dubrulle J, Malapert P, Chal J, Pourquié O (2005). Control of the segmentation process by graded MAPK/ERK activation in the chick embryo. Proc. Natl Acad. Sci. USA.

[11] Aulehla A, Pourquié O (2010). Signaling gradients during paraxial mesoderm development. Cold Spring Harb. Perspect. Biol..

[12] Dubrulle J, Pourquié O (2004). Fgf8 mRNA decay establishes a gradient that couples axial elongation to patterning in the vertebrate embryo. Nature.

[13] Simsek FM, Özbudak EM (2018). Spatial fold change of FGF signaling encodes positional information for segmental determination in zebrafish. Cell Rep..

[14] Regot S, Hughey JJ, Bajar BT, Carrasco S, Covert MW (2014). High-sensitivity measurements of multiple kinase activities in live single cells. Cell.

[15] Mayr V, Sturtzel C, Stadler M, Grissenberger S, Distel M (2018). Fast dynamic in vivo monitoring of Erk activity at single cell resolution in DREKA zebrafish. Front. Cell Dev. Biol..

[16] Akiyama R, Masuda M, Tsuge S, Bessho Y, Matsui T (2014). An anterior limit of FGF/Erk signal activity marks the earliest future somite boundary in zebrafish. Development.

[17] Sari DWK (2018). Time-lapse observation of stepwise regression of Erk activity in zebrafish presomitic mesoderm. Sci. Rep..

[18] Ishimatsu K (2018). Size-reduced embryos reveal a gradient scaling-based mechanism for zebrafish somite formation. Development.

[19] Xu L (2012). Spatio-temporal manipulation of retinoic acid activity in zebrafish hindbrain development via photo-isomerization. Development.

[20] Hamade A (2006). Retinoic acid activates myogenesis in vivo through Fgf8 signalling. Dev. Biol..

[21] Zhang W (2018). Control of protein activity and gene expression by cyclofen-OH uncaging. ChemBioChem.

[22] Yu SR (2009). Fgf8 morphogen gradient forms by a source-sink mechanism with freely diffusing molecules. Nature.

[23] Holley S (2007). The genetics and embryology of zebrafish metamerism. Dev. Dyn..

[24] Schröter C (2012). Topology and dynamics of the zebrafish segmentation clock core circuit. PLoS Biol..

[25] Riedel-Kruse IH, Müller C, Oates AC (2007). Synchrony dynamics during initiation, failure and rescue of the segmentation clock. Science.

[26] Byrd TA (2019). Critical slowing down in biochemical networks with feedback. Phys. Rev. E.

[27] Lopez-Olmeda JF, Sanchez-Vazquez FJ (2011). Thermal biology of zebrafish (Danio rerio). J. Therm. Biol..

[28] Wakamatsu Y, Ogino K, Hirata H (2019). Swimming capability of zebrafish is governed by water temperature, caudal fin length and genetic background. Sci. Rep..

[29] Romney ALT, Davis EM, Corona MM, Wagner JT, Podrabsky JE (2018). Temperature-dependent vitamin D signaling regulates developmental trajectory associated with diapause in an annual killifish. Proc. Natl Acad. Sci. USA.

[30] Westerfield, M. *The Zebrafish Book. A Guide for The Laboratory Use of Zebrafish (Danio rerio)* (The University of Oregon Press, 2000).

[31] Kimmel CB, Ballard WW, Kimmel SR, Ullmann B, Schilling TF (1995). Stages of embryonic development of the zebrafish. Dev. Dyn..

[32] Feng Z (2017). Optical control of tumor induction in the zebrafish. Sci. Rep..

[33] Pfaffl MW, Tichopad A, Prgomet C, Neuvians TP (2004). Determination of stable housekeeping genes, differentially regulated target genes and sample integrity: BestKeeper—Excel-based tool using pair-wise correlations. Biotech. Lett..

[34] Thisse C, Thisse B, Schilling TF, Postlethwait JH (1993). Structure of the zebrafish snail1 gene and its expression in wild-type, spadetail and no tail mutant embryos. Development.

[35] Furthauer M, Thisse C, Thisse B (1997). A role for FGF-8 in the dorsoventral patterning of the zebrafish gastrula. Development.

[36] Deniziak M (2007). Loss of selenoprotein N function causes disruption of muscle architecture in the zebrafish embryo. Exp. Cell Res..

[37] Sinha DK (2010). Photocontrol of protein activity in cultured cells and zebrafish with one- and two-photon illumination. Chembiochem.

[38] Herrgen, L., Schröter, C., Bajard, L., & Oates, A. C. *Zebrafish* 243–254 (Humana Press, 2009). 10.1007/978-1-60327-977-2_15 19378108

