# Supplementary material for: Fgf8 dynamics and critical slowing down may account for the temperature independence of somitogenesis
Source: Commun Biol. 2022 Feb 7;5:113. doi: 10.1038/s42003-022-03053-0 (PMC8821593; doi:10.1038/s42003-022-03053-0)
Supplement: Supplementary file 2 — Supplementary Information [file 42003_2022_3053_MOESM2_ESM.pdf]

SUPPLEMENTARY MATERIAL

Fgf8 dynamics and critical slowing down may account for the temperature independence of somitogenesis

Weiting Zhang[1,2,♦], Pierluigi Scerbo[1,2,♦], Marine Delagrange[1,2,3], Virginie Candat[1,2], Vanessa Mayr[4], Sophie Vriz[5], Martin Distel[4] , Bertrand Ducos[1,2,3,] and David Bensimon[1,2,6,*]*

1. LPENS, PSL, CNRS, 24 rue Lhomond, Paris 75005
2. IBENS, PSL, CNRS, 46 rue d'Ulm, Paris 75005
3. High Throughput qPCR Core Facility, ENS, PSL, 46 rue d'Ulm, Paris 75005
4. St Anna Children's Cancer Research Institute, Zimmermannplatz 10, 1090 Vienna
5. CIRB, Collège de France, Paris 75005
6. Dept. Chemistry and Biochemistry, UCLA, Los Angeles 90094

- ♦ Co-first authors

* Mail to: david@phys.ens.fr or bertrand.ducos@phys.ens.fr

Fig. S1 The Erk boundary domain displays periodic jumps of the size of a somite.

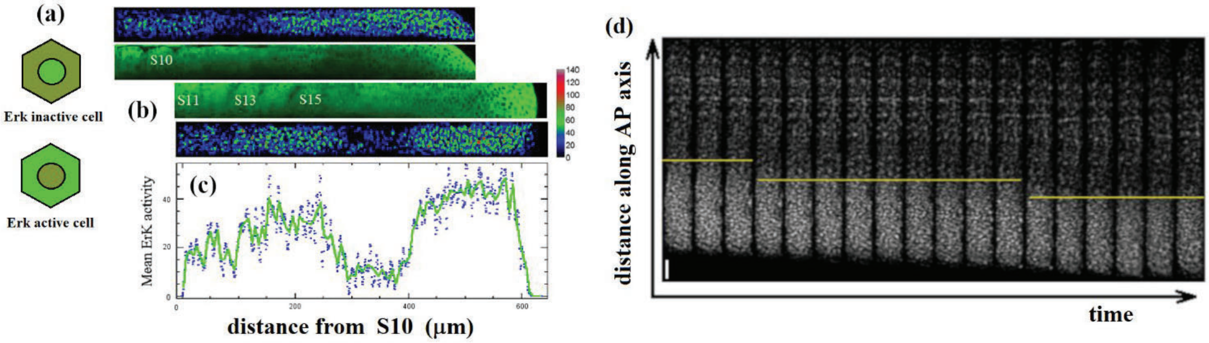

Fig. S1: (a) Schematic illustration of the Erk reporter: localized mostly in the nucleus in inactive cell and in the cytoplasm in active ones (with phosphorylated Erk). b) Confocal image of the Erk activity domain in DREKA embryos (expressing a kinase translocation fluorescent reporter) at 10 and 15 somite stages. Erk activity changes are reflected by changes of the fluorescent contrast between cytoplasm and nucleus[14,15]. The real color images are images in one confocal plane. The false color images represent the Erk activity averaged over a 56 µm perpendicular stack of images. (c) Mean Erk activity at 15 somite stage further averaged over a lateral sliding window of 0.6µm (1 pixel in size, blue dots (raw data)) or 6µm (10 pixels, green curve) along the AP axis. (d) Time lapse observations of the Erk activity domain in DREKA embryos (the fluorescent contrast between nucleus and cytoplasm is displayed here). The time span between images is 5 min. (the period of the clock in this experiment is about 45min.) The yellow lines point to the boundary of Erk activity which periodically jumps by about 50 µm. Scale bar: 50 µm.

Fig. S2 The Erk boundary domain and wavefront velocity is insensitive to perturbations of the RA concentration.

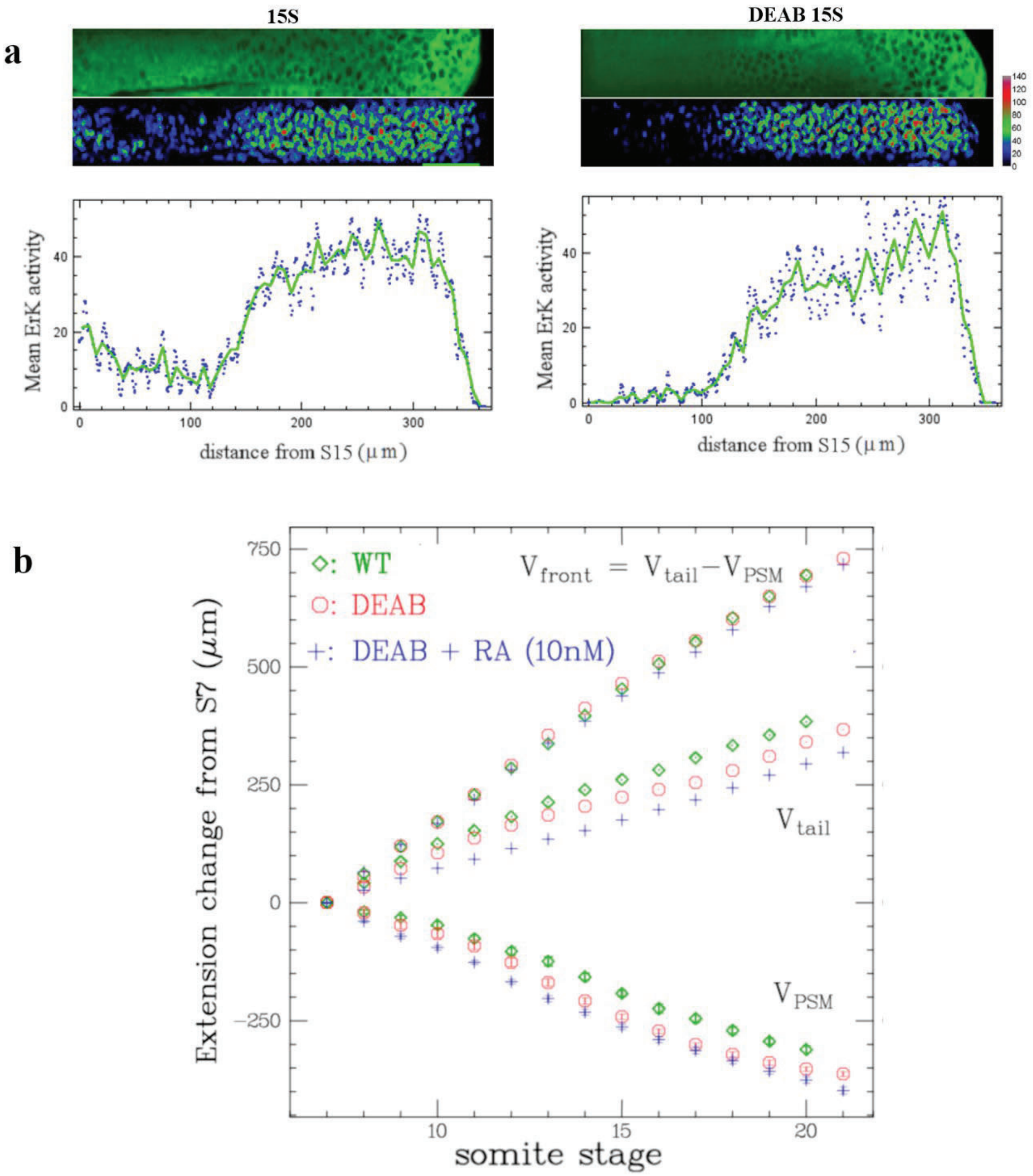

Fig. S2: (a) The Erk activity domain (observed here at 15 somites) in DREKA embryos incubated or not in 10 μM DEAB (from one-cell stage). Real color images are from one confocal plane. The false color images represent the Erk activity averaged over a 56 μm perpendicular stack of images. The bottom plot is the mean Erk activity at 15 somite stage further averaged over a lateral sliding window of 0.6μm (1 pixel in size, blue dots (raw data)) or 6μm (10 pixels, green curve) along the AP axis. Notice that the Erk activity domain is barely affected by incubation in DEAB. (b) Tail growth and PSM shrinkage rates in embryos without DEAB (n=8) and with DEAB without (n=12) and with (n=14) exposure to 10nM external RA (at 70% epiboly). The rates of growth and shrinkage are barely affected by these perturbations.

Fig. S3 RA positively regulates Fgf8.

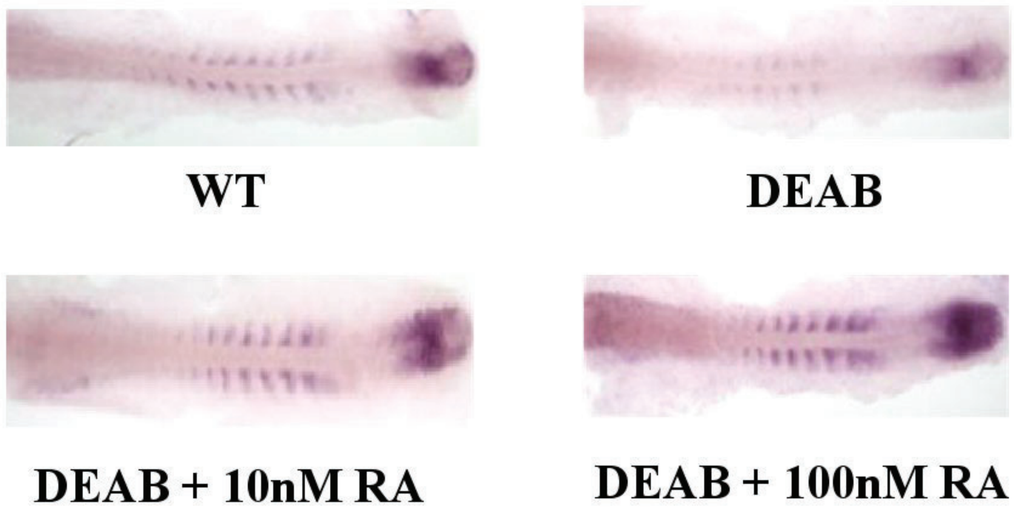

Fig. S3: In-situ hybridization against Fgf8 mRNA in WT embryos and in embryos incubated in 10 μM DEAB (from one cell stage) without or with 5 minute incubation at 90% epiboly in 10 or 100 nM Retinoic acid.

Fig. S4 The Erk activity gradient is similar with and without exogenous Fgf8 activation.

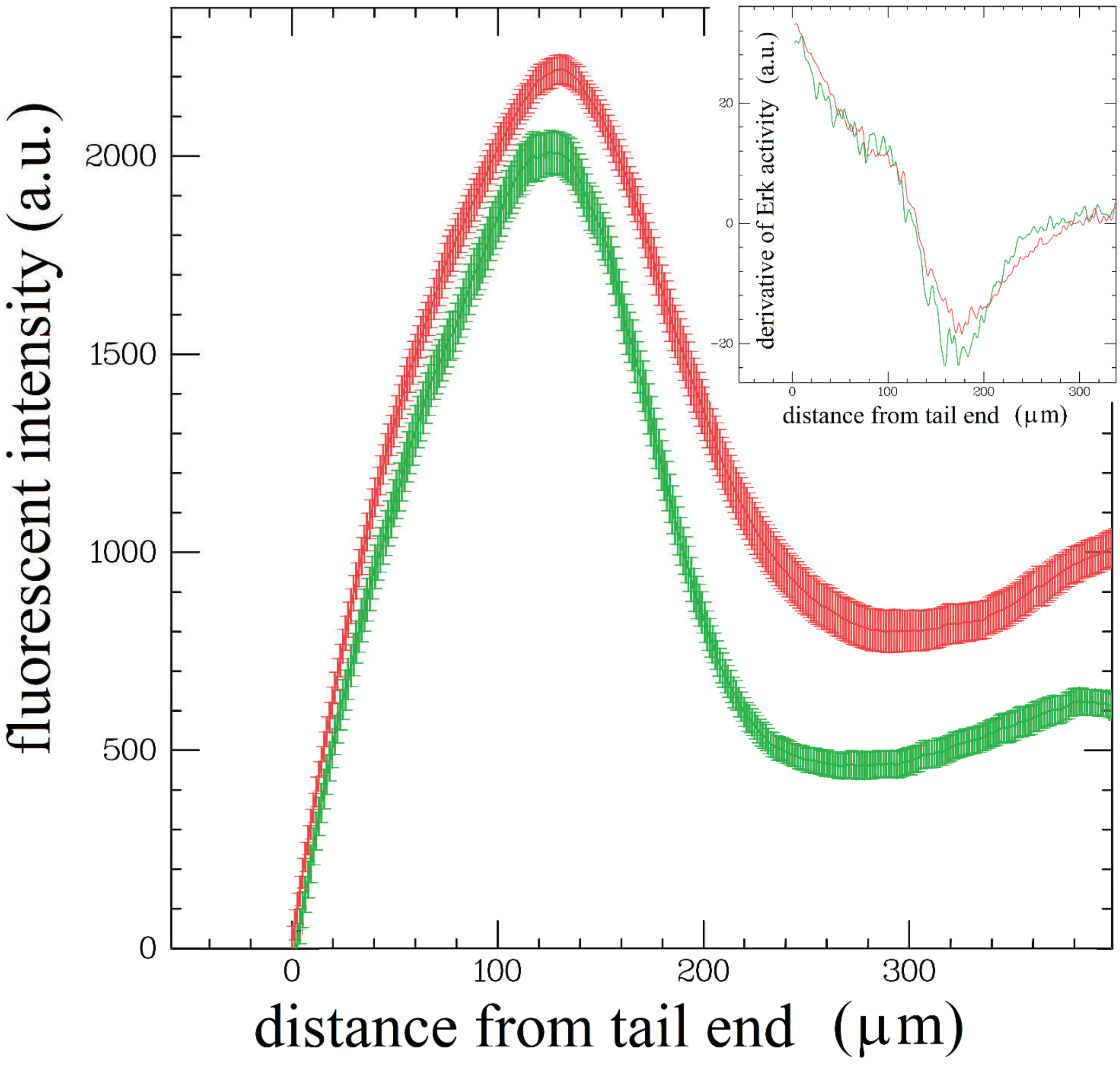

Fig. S4: Mean fluorescence intensity of probes against phosphorylated Erk (reporting on Erk activity) in embryos at 15 somite stage with no activation of exogenous fgf8 (green; n=16) and in embryos where an exogenous source of Fgf8 was activated (red ; n=24). Error bars are standard deviation on the mean. Inset: Derivative of the mean Erk activity signal displaying similar behavior with and without activation of the exogenous Fgf8 source.

Fig. S5 The Erk activity is similar at similar stages of somitogenesis but different temperatures

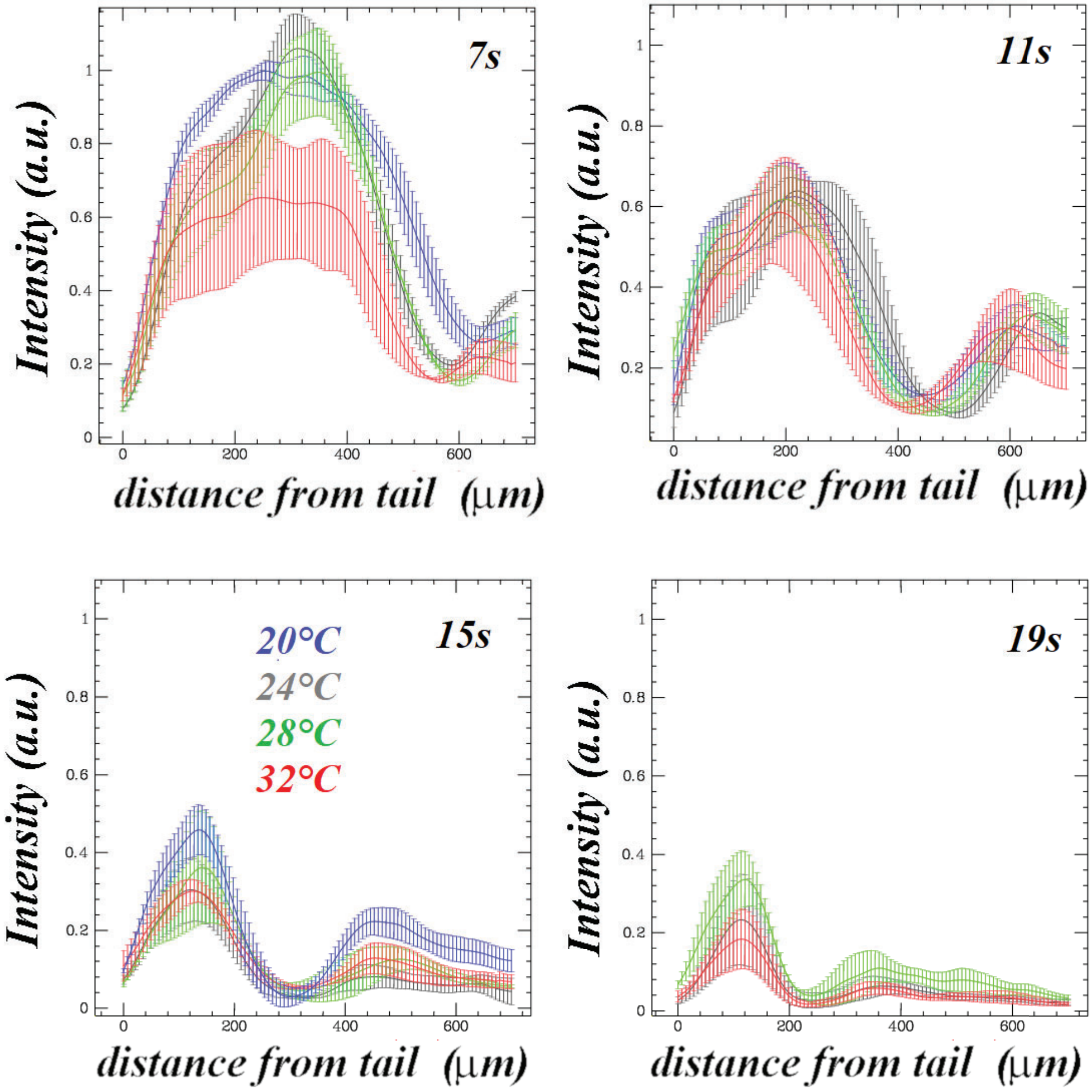

Fig. S5: Phosphorylated Erk concentration (deduced from the IHC fluorescence images, see Fig.5) from the tail end of the developing embryos at similar stages of somitogenesis (7, 11, 15 and 19 somites) and different temperatures (20°C, 24°C, 28°C and 32°C). The fluorescence intensity measured in each embryo within a sliding window of 20 μm is averaged over n=3 embryos. The error bars are errors on the mean estimated from the data on these 3 embryos.

Fig. S6 Differentiation gene transcripts vary with the somite stage.

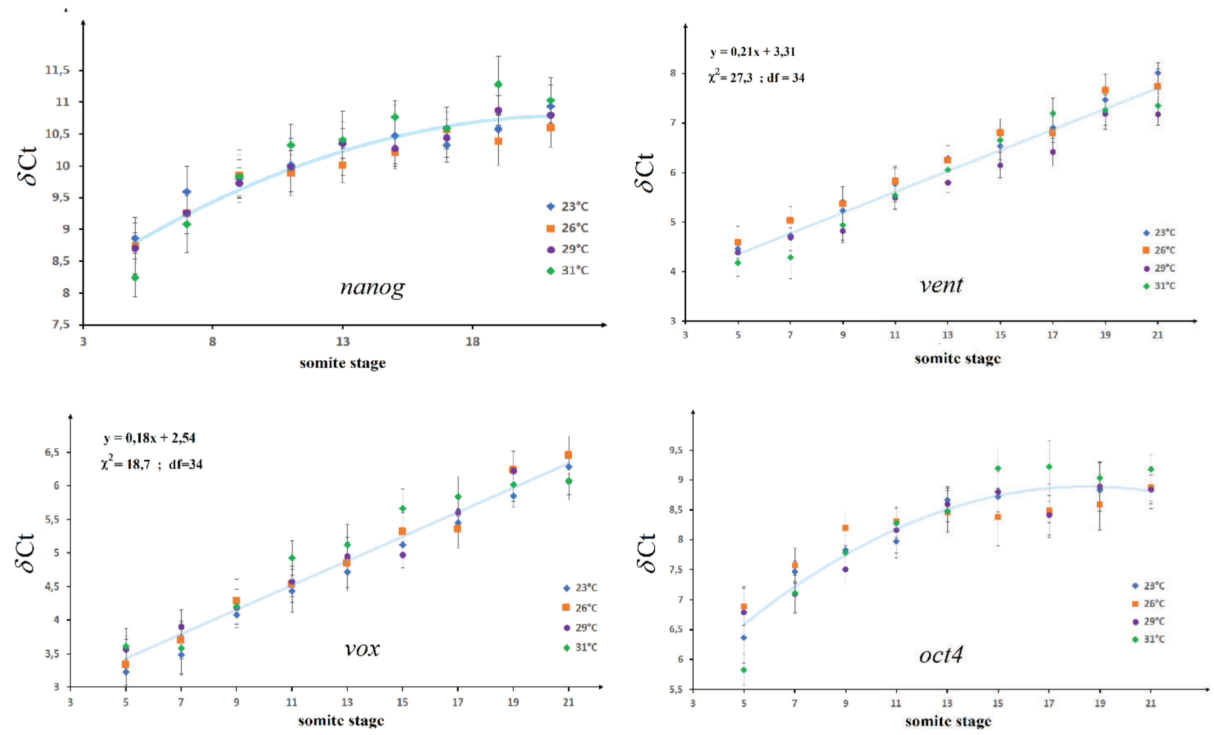

Fig. S6: Variation with temperature of the δCt of various gene transcripts (*nanog, vent, vox, oct4*) known to play a role in cellular differentiation relative to house-keeping genes (*rpl13* or *β-actin*). Notice that as a function of somite stage the data taken at different temperatures collapse on the same curve. For some genes for which δCt appears to vary linearly with somite stage (exponentially decreasing concentrations) we display the best fit. For others the continuous curve is just a guide to the eye.

Fig. S7 The slope of δCt .vs. time. For a number of genes displays critical behavior.

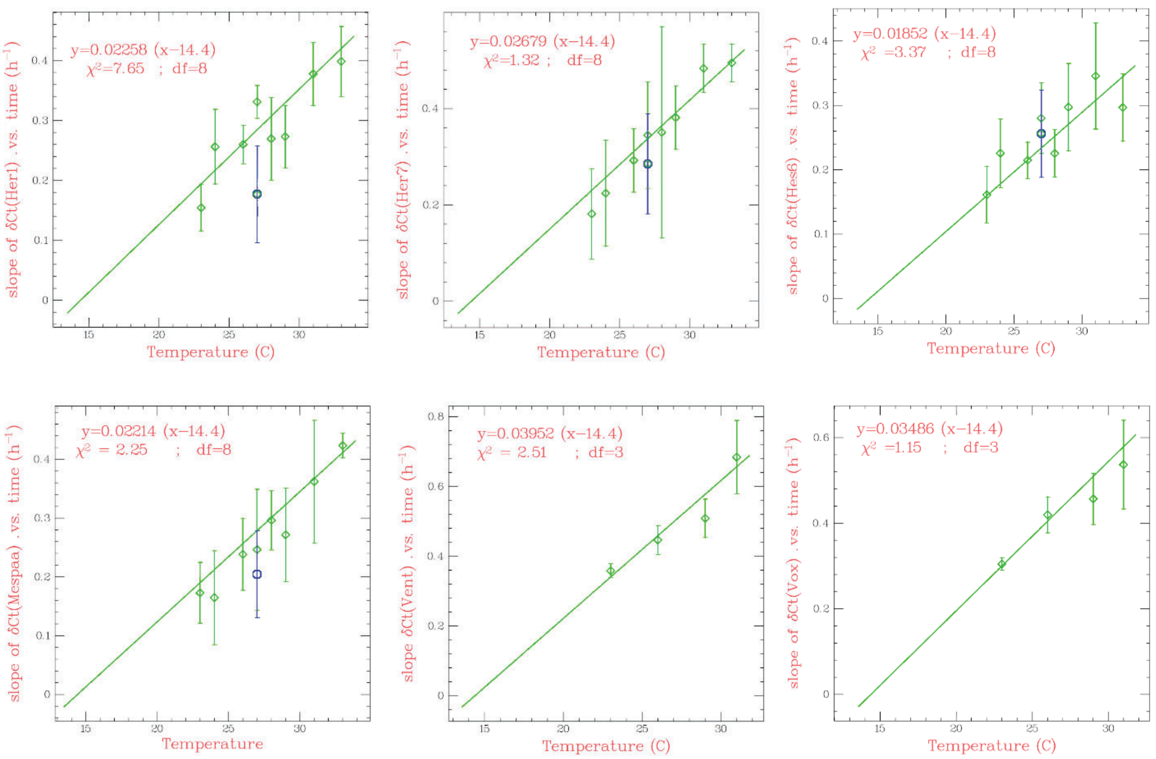

Fig. S7: For a number of genes (*her1, her7, hes6, mespaa, vent, vox*) whose δCt varies linearly with time (exponentially decreasing concentrations, see Fig.7 and S5) we computed the best fit slope y for every temperature (the blue point at 27°C is the best fit slope to PSM only data) . That slope is here plotted as a function of temperature and is fitted to a critical behavior: $y = a(T-Tc)$ with $Tc=14.4$ °C . This analysis confirms the critical behavior of many genes implicated in somitogenesis and cellular differentiation.

Fig. S8 Some genes do not vary during somitogenesis.

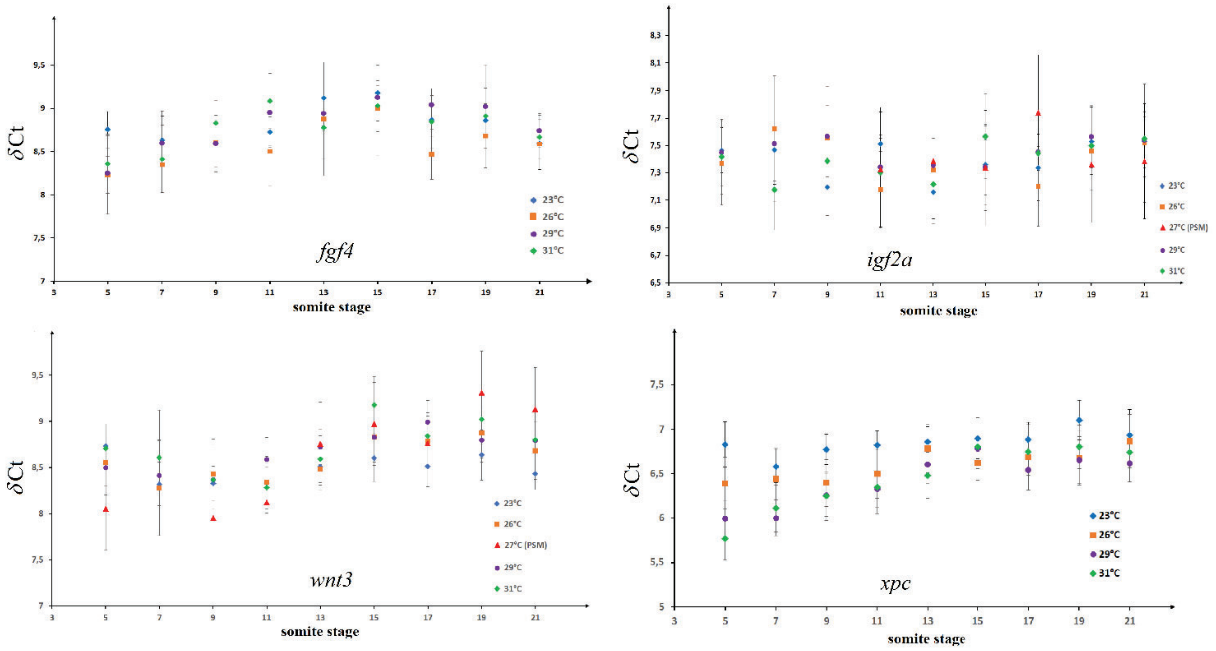

Fig. S8: Some of the gene transcripts we studied (*fgf4, igf2a, wnt3, xpc*) did not appear to vary during somitogenesis (when compared to house-keeping genes such *rpl13* or *β-actin*) and displayed similar relative concentrations at different temperatures.

Fig. S9 The dynamics of Fgf8 in the progenitor region.

Our observations are compatible with a very simple dynamics of the Fgf8 mRNA concentration in the progenitor region ($mF_0(t)$ = [*fgf8m*(*0,t*)]), whereby its time variation (a result of production, dilution upon division and degradation) can be described by the equation:

$$\frac{d\, mF_0}{dt} = -\, a(t) mF_0 \qquad \text{(S1)}$$

Our observations of an exponential decrease of <[*fgf8m*(*x,t*)]> suggest that $a(t) = 1/\tau$.

The same equation can account for the recently published results on somitogenesis in PSM explants[13] if we assume that in these experiments *a(t)* decreased exponentially with time: $a(t) = (a/\tau)\, e^{-t/\tau}$. In that case $mF_0(t) \sim \exp[a\, (e^{-t/\tau} -1)]$ with *a* of *O*(1) and τ a few somatic periods. If a threshold in the local Fgf8 gradient triggers the differentiation into somites, one then expects a linear correlation between PSM and somite size, Fig.S8, as reported.

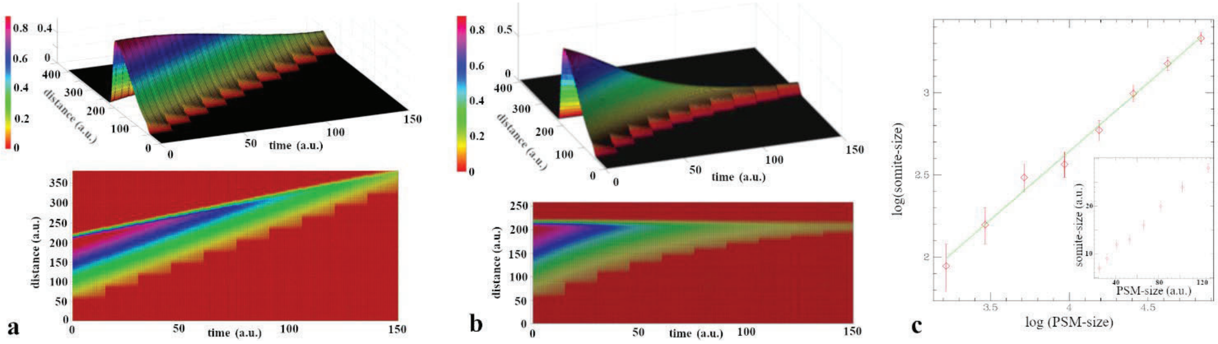

Fig. S9: Variation of Erk activity as a function of time and distance along the embryo in a model where the gradient of Fgf8 decays with time and somites are formed periodically when the Fgf8 gradient falls below a given threshold: (a) for an exponentially decaying gradient of Fgf8 as in the present experiments on a growing embryo: somites have constant size and the PSM catches up with the growing tail; (b) for Fgf8 decaying as in equation S1 in a non-growing tail explant with an exponentially decreasing rate of Fgf8 dilution, a(t): the somite size decreases exponentially with time. (c) somite size vs PSM length in logarithmic scale (inset: linear scale) for the situation simulated in (b) which is consistent with the published data[13].

Supplementary Note 1

A simple model for critical slowing down in cellular metabolism

Let a be the concentration of a component required for the transcription of many genes (such as a particular σ-factor or a component of the RNA-pol complex), $k_a(T)$ its rate of transcription, $d_a + \gamma a$ its rate of degradation. Suppose that there is a temperature T_c where $k_a(T_c)= d_a$. Near that temperature the transcription of a satisfies:

$$\frac{da}{dt} = k_a(T)a - d_a a - \gamma a^2 = k'_a(T_c)(T - T_c)a - \gamma a^2 \qquad \text{(S2)}$$

For $k'_a > 0$, there is a stable solution with $a > 0$ only when $T > T_c$. Defining $a = (T-T_c)\alpha;\ \theta = (T- T_c)t$ yields :

$$\frac{d\alpha}{d\theta} = k'_a(T_c)\alpha - \gamma\alpha^2 \qquad \text{(S3)}$$

Which solution is $\alpha(\theta)=\alpha(t/\tau_a)$ with $\tau_a = 1/[k'_a(T_c)(T - T_c)]$. The dynamics of a displays critical slowing down, i.e. τ_a diverges as $T\rightarrow T_c$.
If the transcription of a gene b depends on protein product A (proportional to a) and its degradation is constant then:

$$\frac{db}{dt} = k_b a - d_b b \qquad \text{(S4)}$$

If $d_b\tau_a >> 1$, b is slaved to a: $b = k_b (T-T_c)\ \alpha(t/\tau_a)/d_b \equiv (T-T_c)\ \beta(t/\tau_a)$. Gene b thus displays critical slowing down, just as gene a.
If the protein product B (proportional to b) is a degrading enzyme for a gene c whose transcription is under feedback from its product C and under control of A, then:

$$\frac{dc}{dt} = k_c(C)a - d_c Bc = k_c(C)a - d'_c bc \qquad \text{(S5)}$$

Where k_c (C) could be a function of c, via its protein product C:

$$\frac{dc}{d\theta} = k_c(C)\alpha - d'_c\beta c \qquad \text{(S6)}$$

In all cases we see that a,b and c display critical slowing down as $T\rightarrow T_c$, with a functional behavior that depends on the values and dependences of $k_c(C)$ and d_c. In particular if $k_c(C) = k'_c c$, the concentration of c varies exponentially with time as observed here for many genes. Similarly, for oscillations resulting from delayed transcriptional/translational feedback (as postulated for the segmentation clock), the fact that the concentration of the critical transcriptional component a varies as $T-T_c$ results in a transcriptional delay that increases as $1/(T-T_c)$ and a consequent period of oscillations that diverges as $T\rightarrow T_c$.

The simple scenario sketched above for the critical slowing down of some cellular gene networks is based on a competition between transcriptional feedback and degradation of a key transcriptional component of the network a. Exactly analogous arguments can be made with a gene network based on phosphorylation/dephosphorylation reactions controlled by a common factor, a. In other words, there are many possible scenarios[26] accounting for a critical slowing down of cellular networks and it is too early to judge which one would best account for the critical slowing down observed during zebrafish somitogenesis and development.

Table S1: List of the hydrolysis probes used to target the genes studied

Target genes	Taqman® Reference
actb1	Dr03432610_m1
Aldh1a2	Dr03131678_m1
Axin2	Dr03074141_m1
bactin	1358731A6
Bmp4	Dr03118800_m1
Crabp1a	Dr03112659_mH
Crabp1b	Dr03070892_m1
Crabp2a	Dr03119163_m1
Ctnnb1	Dr03432627_m1
Cyp26a1	Dr03086662_m1
DeltaC	Dr03073935_m1
DeltaD	Dr03111905_m1
Dusp6	Dr03150653_g1
Fgf17	Dr03101579_m1
Fgf3	Dr03432794_m1
Fgf4	Dr03080033_m1
Fgf8a	Dr03105657_m1
Fgf8b	Dr03433600_m1
Fgfr4	Dr03131500_m1
Foxa3	Dr03125016_m1
Her1	Dr03124981_m1
Her5	Dr03150143_g1
Her7	Dr03125132_g1
Hes6	Dr03131925_g1
Hnf1bb	Dr03144482_m1
Hoxa1a	Dr03138352_g1
Hoxa5a	Dr03138353_m1
Hoxb1b	Dr03432669_g1
Hoxd4a	Dr03432559_m1
Ifg1rb	Dr03112490_m1
Igf1	Dr03105961_m1
Igf1ra	Dr03106066_m1
Igf2a	Dr03150335_m1
Mespaa	Dr03099335_m1
Mespba	Dr03138364_m1

Myf5	Dr03074150_m1
Myod1	Dr03138243_g1
Myog	Dr03138081_m1
nanog	Dr03124475_g1
Notch1A	Dr03112159_m1
Pax6a	Dr03432808_m1
Pax6b	Dr03125140_m1
pou5f3 (zf oct4)	Dr03150184_m1
Rarab	Dr03150327_m1
Rpl13a1	Dr03119261_m1
Rpl13a2	Dr03119263_m1
Tbx24	Dr03433378_m1
Tbx6	Dr03079903_m1
vent	Dr03080182_m1
vox	Dr03433118_m1
Wnt1	Dr03432570_m1
Wnt2	Dr03099103_m1
Wnt3	Dr03435826_m1
