# Supplementary material for: Fgf8 dynamics and critical slowing down may account for the temperature independence of somitogenesis
Source: Commun Biol. 2022 Feb 7;5:113. doi: 10.1038/s42003-022-03053-0 (PMC8821593; doi:10.1038/s42003-022-03053-0)
Supplement: Supplementary file 3 — Description of Additional Supplementary Files [file 42003_2022_3053_MOESM3_ESM.pdf]

Description of Additional Supplementary Files

File name: Supplementary Data 1

Description: Raw data in Excel file (12 sheets)

1. **Erk-7s:** Data at 7 somites and different temperatures used in Fig.S5 _ digitisation of Erk activity from data as in Fig. 7.

2. **Erk-11s:** Data at 11 somites and different temperatures used in Fig.S5 _ digitisation of Erk activity from data as in Fig. 7.

3. **Erk-15s:** Data at 15 somites and different temperatures used in Fig.S5 _ digitisation of Erk activity from data as in Fig. 7.

4. **Erk-19s:** Data at 19 somites and different temperatures used in Fig.S5 _ digitisation of Erk activity from data as in Fig. 7.

5. **RTqPCR_Fgf8_27C:** Data on Fgf8 expression at 27°C vs Rpl13 and b-actin used in Fig.5 _ Obtained on LightCycler 480.

6. **RTqPCR_27C:** Data on gene expression in PSM, remaining Trunk (T) or Whole (W) embryo at 27°C used in Figs. 5, 6, and 8 and Fig. S6.

7. **RTqPCR_stage:** Data on gene expression in Whole embryo at given stage of somitogenesis and temperatures: 23°, 26°, 29° and 31°C used in Figs .6 and 8 and Figs. S6 and S8.

8. **RTqPCR_time:** Data on gene expression in Whole embryo at given time (1-8h) after bud stage and temperatures: 24°, 28° and 33°C used in Figs. 6 and 8.

9. **WT-growth:** Data on growth of tail and shrinkage of PSM in WT embryos used in Fig. 4e and S2.

10. **Fgf_Phenotypes-growth:** Data on growth of tail and shrinkage of PSM in Fgf8 overexpressing embryos used in Fig. 4e.

11. **RA_Phenotype-growth:** Data on growth of tail and shrinkage of PSM in embryos incubated in DEAB and RA used in Fig. S2b.

12. **Erk-with-exoFgf (15s):** Data on Erk activity in WT embryos and embryos overexpressing Fgf8 from an exogenous source (shown in Fig. 4); data used in Fig. S4.
