# Supplementary material for: Fgf8 dynamics and critical slowing down may account for the temperature independence of somitogenesis
Source: Commun Biol. 2022 Feb 7;5:113. doi: 10.1038/s42003-022-03053-0 (PMC8821593; doi:10.1038/s42003-022-03053-0)
Supplement: Supplementary file 1 — Transparent Peer Review File [file 42003_2022_3053_MOESM1_ESM.pdf]

nature portfolio

Peer Review File

Fgf8 dynamics and critical slowing down may account for the temperature independence of somitogenesis

Open Access This file is licensed under a Creative Commons Attribution 4.0 International License, which permits use, sharing, adaptation, distribution and reproduction in any medium or format, as long as you give appropriate credit to the original author(s) and the source, provide a link to the Creative Commons license, and indicate if changes were made. In the cases where the authors are anonymous, such as is the case for the reports of anonymous peer reviewers, author attribution should be to 'Anonymous Referee' followed by a clear attribution to the source work. The images or other third party material in this file are included in the article's Creative Commons license, unless indicated otherwise in a credit line to the material. If material is not included in the article's Creative Commons license and your intended use is not permitted by statutory regulation or exceeds the permitted use, you will need to obtain permission directly from the copyright holder. To view a copy of this license, visit http://creativecommons.org/licenses/by/4.0/.

Reviewers' comments:

Reviewer #1 (Remarks to the Author):

This is a nice paper that studies somitogenesis in embryos of zebrafish. In particular the temperature dependence (or rather independence) is investigated.

I am not an experimentalist working in the field so I concentrate more on the presentation of the general results. I find that the paper is a nice contribution to enlighten details of somitogenesis. My main point however is that I am not so sure which of the observed properties have been reported before and which have not.

The authors find that it is the gradient of Fgf and not the absolute concentration that sets the position of the of somite wavefront. This is surely interesting but - not being an expert in the field - it seems that this has actually been published before.

I further find the results on the temperature dependence quite interesting as indeed it turns out that many properties such as the dynamics of fgf, tail growth, segmentation period etc are strongly temperature dependent. However it seems to me that some of these observations have also been made beforehand by other groups.

So I think the main results can be 'localized' to two main topics. Maybe most important is that most quantities, like decay
times, rates etc follow a scaling with a critical exponent equal to 1 and with a critical temperature of 14.4 oC. In other words the behavior acts like a critical systems. A quite interesting observation although may also raise some criticism as I come to below.

Related to this are the observations that the gene expressions of
the important genes involved on somitogenesis (these are among others her1, hes6, deltaC etc.) follow a critical slowing down again like a critical systems near Tc. This means that the variation of the genes as a function of somite stage should be temperature independent. This I find very interesting and informative and is all nicely 'collapsed' in Fig. 7
From a physics perspective these observations constitute a new way to look at somitogenesis. I wonder however what it actually means biologically? This is discussed vary sparsely and in particular since embryos die below 19 oC as the authors also mention. So what is the meaning with a critical temperature
at Tc=14.4. oC? It is all argued with scaling plots as we are used to in physics. But maybe some of the depicted scalings are not that convincing , like in Fig. 6a. I other words I would urge the authors to present a more elaborate discussion on the biological relevance of somitogenesis being a critical system. And what is the role of a critical temperature.

The notations of the scaling plot like \deltaCt(fgf8(t)) are somewhat confusing. Maybe it just have to do with the fonds but it is difficult to read (and understand).
It might also help with references to equations numbers

There are several small misprints. Please correct them.

I find the paper interesting. It could be published after revisions suggested in the report.

Reviewer #2 (Remarks to the Author):

In this manuscript the authors address two points, i.e. the role of Fgf8/Erk signaling in setting the

wavefront in zebrafish segmentation and second, they address the mechanism related to temperature compensation in zebrafish embryos, resulting in temperature invariant morphology despite temperature dependent change in gene expression dynamics. In my view, the first part, the role of Erk gradient in segmentation is not providing key novel insight (compared to previous reports, such as Simsek et al. 2018) and also, I see several issues that need clarification (see points 1 and 2 below). In the second part, the proposal that segmentation is a system operating close to criticality and the discussion how this is linked to temperature compensation is original, but results and their presentation appears premature. I make suggestions and comments below (4-6).

1) To test the role of Fgf8 and its downstream effector Erk, the authors overexpress Fgf8 ubiquitously, overriding the in vivo gradient of Fgf8 in the PSM. While the authors argue that Fgf/Erk activity is "still stronger at the posterior PSM, Fig.4c." to me, this crucial point is not visible in the data shown. Even if a (slight) gradient was still present in the Fgf8 GOF situation, this would a. need to be quantified and presented and b. this gradient would appear different, in terms of its spatial derivative, compared to control conditions. Given this finding, I do not see how the authors come to this conclusion: this " implies that the somitogenetic wavefront is sensitive to the spatial gradient of Fg8, not its local concentration". Again, the spatial gradient appears to be heavily disrupted in the Fgf8 GOF condition, yet, the authors report that both the wavefront and PSM shrinking rate is not affected. This needs clarification I think. As said above, a rigorous quantification of the Erk gradient slopes is needed here and the interpretations then re-evaluated.
2) The finding of exponential decrease of Fgf8 mRNA levels over time does not, in my view, allow to conclude about how the spatial Fgf8 gradient is changing over time. The authors determine the mean PSM concentration from the entire embryo or entire PSM and hence any spatial information is lost. It would be key to have the information about the slope and amplitude of the spatial Fgf8 gradient spanning the PSM. Given that the authors have an ERK reporter at hand, the quantification of the Erk gradient over ontogenetic time seems feasible.

To me, this part of the paper does not provide convincing/novel insight into the role of Fgf/ERK in segmentation process, compared to previous reports (Simsek et al . 2018).

3) One key finding presented in the second part of the paper is that the timescale at which Fgf8 concentration changes over time matches the changes seen at the level of PSM shrinkage, even across a temperature range. The characterization of timescales is an interesting approach and indeed the system might share features of a system operating in vicinity of criticality. However, as stated also in point 2, while we learn about Fgf8 decay over developmental time at different temperatures, it remains unclear how the spatial Fgf8 gradient changes when changing temperature. For instance, what happens to the slope and amplitude of the spatial Fgf8 gradient (not the temporal one)? The spatial PSM gradient slope is a function of Fgf8 degradation, while only in the tail bud de novo transcription takes place. Hence, how temperature affects Fgf8 gradient, i.e. amplitude and slope, is unclear. In contrast, the Fgf8 decay over developmental time was analyzed as an average without spatial resolution, in all PSM. Without knowing how the gradient changes upon different temperatures, it is not clear whether the changes of Fgf8 can compensate for changes seen at the level of segmentation clock dynamics. As above, since Erk readout is available, with these additional quantification at hand, the hypothesis of a critical system should be re-evaluated.

Reviewer #3 (Remarks to the Author):

In this paper, the authors studied the mechanism of how zebrafish embryos generate regular sizes of somites in a temperature independent manner. To address this issue, they first analyzed the roles of Fgf signaling pathway in somitogenesis by using transgenic zebrafish lines and inhibitors and re-discovered critical roles of spatial gradient of Fgf8 in the determination of wave-fronts. The authors

next employed RT-qPCR and observed that mRNA concentration of Fgf8 and many other genes decreased during somitogenesis. They found that inverse of the decay time (slope of δCt(fgf8)) was proportional to T-T_c ranging from 23 to 31°C with T_c = 14.4°C. Importantly, the velocity of PSM-shrinkage was also inversely proportional to T-T_c, and the ratio between fgf8 decay time and the velocity of PSM-shrinkage was conserved within temperatures; these results indicated the mechanism of how sizes of somites are temperature-compensated.

The authors proposed an attractive hypothesis that could explain the mechanism of robust patterning in somitogenesis in the presence of temperature fluctuation, with referring the concept "critical slowing down" known in the field of physics that explains universal behaviors of dynamical systems near the critical transition point. However, although the data were mostly consistent with previous studies, their novelty is rather limited. In addition, I am concerned about the way of data analysis that is related to the authors' main claims, including the analysis of patterns of Fgf activity.

Major comments:
1) The authors utilized RT-qPCR analysis to evaluate dynamics of Fgf gradient, but the values are relative numbers to reference genes, such as rpl13a and beta-actin whose absolute numbers are unknown. Can the authors rule out the possibility that the observed decay of Fgf8 mRNA is due to the decreased number of Fgf8 expressing cells? It is also unclear whether the observed Fgf8 mRNA dynamics can fully account for the dynamics of the downstream activity, such as the abundances of pErk. Imaging of Erk activity in DREKA embryos or IHC of pErk patterns with different temperature conditions could answer these concerns.

2) In Figs. 6b and 7, the authors merged the data at all temperatures for curve (line) fitting, but statistical comparisons of curves between temperatures are required; it can be statistically tested whether the regression coefficients between different temperatures are indistinguishable or not. This kind of statistical analysis was used in Lauschke et al., 2013 (Nature, 493, pages101–105 (2013).; see Fig.3).

3) According to the inset of Fig. 6a, embryos are viable at 20°C, although the authors mentioned that embryos die below about 19°C. Additional data recorded at 20°C, especially the data of growth/shrinkage rate shown in Fig.6c, are necessary to support the scaling behaviors.

Minor comments:
1) The authors used transgenic zebrafish (DREKA) embryos that carry a fluorescent Erk-activity reporter (Erk-KTR), which was reported by Regot et al., 2014. The original paper of Erk-KTR (Cell 157, 1724 (2014).) should be cited. In addition, appending of schematic illustration explaining how Erk-KTR works (e.g. Fig.1B in Mayr et al., 2018) will help readers.

2) In Fig. 4, the authors used Gal4-ERT system for exogenous expression of Fgf8 with cyclofen. Were the wild-type embryos also treated with cyclofen? If not, the data of negative control is necessary.

3) Why are two points present at 27°C in Fig. 6a?

Reviewers' comments:

Reviewer #1 (Remarks to the Author):

This is a nice paper that studies somitogenesis in embryos of zebrafish. In particular the temperature dependence (or rather independence) is investigated.

I am not an experimentalist working in the field so I concentrate more on the presentation of the general results. I find that the paper is a nice contribution to enlighten details of somitogenesis. My main point however is that I am not so sure which of the observed properties have been reported before and which have not.

First, we thank this reviewer for his positive remarks on this work which he/she finds to be a “nice contribution to enlighten details of somitogenesis”.

The authors find that it is the gradient of Fgf and not the absolute concentration that sets the position of the of somite wavefront. This is surely interesting but - not being an expert in the field - it seems that this has actually been published before.

I further find the results on the temperature dependence quite interesting as indeed it turns out that many properties such as the dynamics of fgf, tail growth, segmentation period etc are strongly temperature dependent. However it seems to me that some of these observations have also been made beforehand by other groups.

The main concern of this referee regards those findings that were previously reported and those that are new. Indeed, the possible dependence of somitogenesis on the gradient of Fgf8 was previously reported (Simsek and Özbudak, 2018), as mentioned at numerous places in our manuscript. These experiments with tail explants are different from the ones presented here and thus we think that our observations on live embryos are worth reporting. They support and quantitate the point of view mentioned in this previous work.

As for the observation of critical slowing down, this -to our knowledge- was not made before, even though the temperature dependence of the clock was studied earlier (Schröter et al., 2008), as mentioned in our manuscript at many places.

So I think the main results can be 'localized' to two main topics. Maybe most important is that most quantities, like decay times, rates etc follow a scaling with a critical exponent equal to 1 and with a critical temperature of 14.4 oC. In other words the behavior acts like a critical systems. A quite interesting observation although may also raise some criticism as I come to below.

Related to this are the observations that the gene expressions of the important genes involved on somitogenesis (these are among others her1, hes6, deltaC etc.) follow a critical slowing down again like a critical systems near Tc. This means that the variation of the genes as a function of somite stage should be temperature independent. This I find very interesting and informative and is all nicely 'collapsed' in Fig. 7.
From a physics perspective these observations constitute a new way to look at somitogenesis. I wonder however what it actually means biologically? This is discussed vary sparsely and in particular

since embryos die below 19 oC as the authors also mention. So what is the meaning with a critical temperature
at Tc=14.4. oC? It is all argued with scaling plots as we are used to in physics. But maybe some of the depicted scalings are not that convincing , like in Fig. 6a. I other words I would urge the authors to present a more elaborate discussion on the biological relevance of somitogenesis being a critical system. And what is the role of a critical temperature.

Concerning the possible biological meaning of the critical temperature, we have added a paragraph where we discuss that issue. In particular, it appears that the critical temperature measured here is close to the reported temperature where the adult fish metabolic pathways seem to shut down affecting its swimming and survival capacity (Wakamatsu et al., 2019, Lopez-Olmeda et al., 2010). Our observation may also be related to the phenomenon of diapause (Romney et al., 2018): at certain developmental stages (e.g. mid-somitogenesis) and under certain environmental conditions (such as too high or too low temperatures) the development of certain fish may enter a dormancy (or pausing) state, which might correspond to the sub-critical phase which may not be lethal in those species.

The notations of the scaling plot like \deltaCt(fgf8(t)) are somewhat confusing. Maybe it just have to do with the fonds but it is difficult to read (and understand).
It might also help with references to equations numbers

Done.

There are several small misprints. Please correct them.

Done, hopefully….

I find the paper interesting. It could be published after revisions suggested in the report.

Reviewer #2 (Remarks to the Author):

In this manuscript the authors address two points, i.e. the role of Fgf8/Erk signaling in setting the wavefront in zebrafish segmentation and second, they address the mechanism related to temperature compensation in zebrafish embryos, resulting in temperature invariant morphology despite temperature dependent change in gene expression dynamics. In my view, the first part, the role of Erk gradient in segmentation is not providing key novel insight (compared to previous reports, such as Simsek et al. 2018) and also, I see several issues that need clarification (see points 1 and 2 below). In the second part, the proposal that segmentation is a system operating close to criticality and the discussion how this is linked to temperature compensation is original, but results and their presentation appears premature. I make suggestions and comments below (4-6).

As mentioned above we agree with this reviewer that the finding about the role of the Erk gradient during segmentation is not novel, though the particular experiment testing that role here is different from the one previously reported (Simsek and Özbudak, 2018). Concerning his specific comments:

1) To test the role of Fgf8 and its downstream effector Erk, the authors overexpress Fgf8 ubiquitously, overriding the in vivo gradient of Fgf8 in the PSM. While the authors argue that Fgf/Erk activity is

”still stronger at the posterior PSM, Fig.4c.” to me, this crucial point is not visible in the data shown. Even if a (slight) gradient was still present in the Fgf8 GOF situation, this would a. need to be quantified and presented and b. this gradient would appear different, in terms of its spatial derivative, compared to control conditions. Given this finding, I do not see how the authors come to this conclusion: this “ implies that the somitogenetic wavefront is sensitive to the spatial gradient of Fg8, not its local concentration”. Again, the spatial gradient appears to be heavily disrupted in the Fgf8 GOF condition, yet, the authors report that both the wavefront and PSM shrinking rate is not affected. This needs clarification I think. As said above, a rigorous quantification of the Erk gradient slopes is needed here and the interpretations then re-evaluated.

The gradient of Erk activity has been quantified in the GOF experiments and are presented in the revised version, Fig.S4. One notices the good agreement between the shape of the gradient with or without global expression of exogenous Fgf8, thus supporting our claim that the gradient of Fgf8 and not its concentration is the determining factor (a conclusion already reported in Simsek et al, 2018).

2) The finding of exponential decrease of Fgf8 mRNA levels over time does not, in my view, allow to conclude about how the spatial Fgf8 gradient is changing over time. The authors determine the mean PSM concentration from the entire embryo or entire PSM and hence any spatial information is lost. It would be key to have the information about the slope and amplitude of the spatial Fgf8 gradient spanning the PSM. Given that the authors have an ERK reporter at hand, the quantification of the Erk gradient over ontogenetic time seems feasible. To me, this part of the paper does not provide convincing/novel insight into the role of Fgf/ERK in segmentation process, compared to previous reports (Simsek et al . 2018).

To better support the claim that the Fgf8 gradient is changing with somite stage yet independent of temperature we have measured the Erk activity with IHC staining at different developmental stages and different temperatures. The data presented in Figs.7 and S5 support our previous findings. However, we agree with that reviewer that the role of the Fgf8 gradient has already been studied in (Simsek and Özbudak, 2018), albeit in a non-native situation (tail explants) that results in somite with decreasing length.

3) One key finding presented in the second part of the paper is that the timescale at which Fgf8 concentration changes over time matches the changes seen at the level of PSM shrinkage, even across a temperature range. The characterization of timescales is an interesting approach and indeed the system might share features of a system operating in vicinity of criticality. However, as stated also in point 2, while we learn about Fgf8 decay over developmental time at different temperatures, it remains unclear how the spatial Fgf8 gradient changes when changing temperature. For instance, what happens to the slope and amplitude of the spatial Fgf8 gradient (not the temporal one)? The spatial PSM gradient slope is a function of Fgf8 degradation, while only in the tail bud de novo transcription takes place. Hence, how temperature affects Fgf8 gradient, i.e. amplitude and slope, is unclear. In contrast, the Fgf8 decay over developmental time was analyzed as an average without spatial resolution, in all PSM. Without knowing how the gradient changes upon different temperatures, it is not clear whether the changes of Fgf8 can compensate for changes seen at the level of segmentation clock dynamics. As above, since Erk readout is available, with these additional quantification at hand, the hypothesis of a critical system should be re-evaluated.

As explained above we have quantified the Erk activity gradient not only as a function of developmental stage but also as a function of temperature. The data shows that the spatial variation

of Erk activity depends on the stage of somitogenesis but is independent of the temperature. These novel results support our interpretation of the slowing down of somitogenesis as a system near a critical point.

Reviewer #3 (Remarks to the Author):

In this paper, the authors studied the mechanism of how zebrafish embryos generate regular sizes of somites in a temperature independent manner. To address this issue, they first analyzed the roles of Fgf signaling pathway in somitogenesis by using transgenic zebrafish lines and inhibitors and re-discovered critical roles of spatial gradient of Fgf8 in the determination of wave-fronts. The authors next employed RT-qPCR and observed that mRNA concentration of Fgf8 and many other genes decreased during somitogenesis. They found that inverse of the decay time (slope of δCt(fgf8)) was proportional to T-T_c ranging from 23 to 31°C with T_c = 14.4°C. Importantly, the velocity of PSM-shrinkage was also inversely proportional to T-T_c, and the ratio between fgf8 decay time and the velocity of PSM-shrinkage was conserved within temperatures; these results indicated the mechanism of how sizes of somites are temperature-compensated.

The authors proposed an attractive hypothesis that could explain the mechanism of robust patterning in somitogenesis in the presence of temperature fluctuation, with referring the concept "critical slowing down" known in the field of physics that explains universal behaviors of dynamical systems near the critical transition point. However, although the data were mostly consistent with previous studies, their novelty is rather limited. In addition, I am concerned about the way of data analysis that is related to the authors' main claims, including the analysis of patterns of Fgf activity.

We agree with this reviewer that some of our findings concerning the role of the Fgf8 gradient during somitogenesis have been previously reported (Simsek and Özbudak, 2018), albeit in a different non-native situation (tail explants). As a result, the somite size in these experiments shrinks with time instead of being constant as observed in natural conditions. The connection between the temperature independence of somitogenesis and critical phenomena is however new (to the best of our knowledge). In answer to his/her specific comments:

Major comments:
1) The authors utilized RT-qPCR analysis to evaluate dynamics of Fgf gradient, but the values are relative numbers to reference genes, such as rpl13a and beta-actin whose absolute numbers are unknown. Can the authors rule out the possibility that the observed decay of Fgf8 mRNA is due to the decreased number of Fgf8 expressing cells? It is also unclear whether the observed Fgf8 mRNA dynamics can fully account for the dynamics of the downstream activity, such as the abundances of pErk. Imaging of Erk activity in DREKA embryos or IHC of pErk patterns with different temperature conditions could answer these concerns.

Concerning the remark that Fgf8 mRNA decay could be due to decreasing number of expressing cells, this is certainly a possibility compatible with our finding, but which we do not address in this paper. Concerning the reviewer's suggestion that we image Erk activity during somitogenesis, we have conducted the suggested IHC on Erk activity at different stages and temperatures. These data, presented in Fig.7 and S5, confirm our previous findings and should hopefully answer the concerns of this reviewer.

2) In Figs. 6b and 7, the authors merged the data at all temperatures for curve (line) fitting, but statistical comparisons of curves between temperatures are required; it can be statistically tested whether the regression coefficients between different temperatures are indistinguishable or not. This kind of statistical analysis was used in Lauschke et al., 2013 (Nature, 493, pages101–105 (2013).; see Fig.3).

The suggested statistical analysis of the regression curves at different temperatures has been conducted and is presented in Fig.S7. It confirms our previous analysis based on the collapse of the curves taken at different temperatures.

3) According to the inset of Fig. 6a, embryos are viable at 20°C, although the authors mentioned that embryos die below about 19°C. Additional data recorded at 20°C, especially the data of growth/shrinkage rate shown in Fig.6c, are necessary to support the scaling behaviors.

As requested by this reviewer, data on embryo development and Erk activity at 20°C was taken and compared to higher temperatures. The data fits the overall picture of the similarity of growth and Erk activity at given somite stages independent of temperature, Fig.7 and S5. These novel data support our interpretation of somitogenesis as a phenomenon near a critical point.

Minor comments:
1) The authors used transgenic zebrafish (DREKA) embryos that carry a fluorescent Erk-activity reporter (Erk-KTR), which was reported by Regot et al., 2014. The original paper of Erk-KTR (Cell 157, 1724 (2014).) should be cited. In addition, appending of schematic illustration explaining how Erk-KTR works (e.g. Fig.1B in Mayr et al., 2018) will help readers.

The paper of Regot et al. on the original Erk-KTR reporter is cited and a schematic illustration of its working principle is presented in Fig.S1(a).

2) In Fig. 4, the authors used Gal4-ERT system for exogenous expression of Fgf8 with cyclofen. Were the wild-type embryos also treated with cyclofen? If not, the data of negative control is necessary.

No effect of cyclofen on WT embryos was observed.

3) Why are two points present at 27°C in Fig. 6a?

The two points at 27°C correspond to data on whole embryo extract or just tail extracts (PSM only).

REVIEWERS' COMMENTS:

Reviewer #1 (Remarks to the Author):

In my opinion the authors have convincingly responded to the long list of suggestions and criticisms from the three referees. I would suggest publications of the revised manuscript.

Reviewer #3 (Remarks to the Author):

The authors properly answered my comments, and I now support the acceptance of the paper.
